# Inhibition of the glutamine transporter SNAT1 confers neuroprotection in mice by modulating the mTOR-autophagy system

**DOI:** 10.1038/s42003-019-0582-4

**Published:** 2019-09-18

**Authors:** Daisuke Yamada, Kenji Kawabe, Ikue Tosa, Shunpei Tsukamoto, Ryota Nakazato, Miki Kou, Koichi Fujikawa, Saki Nakamura, Mitsuaki Ono, Toshitaka Oohashi, Mari Kaneko, Shioi Go, Eiichi Hinoi, Yukio Yoneda, Takeshi Takarada

**Affiliations:** 1 Department of Regenerative Science, Okayama University Graduate School of Medicine, Dentistry and Pharmaceutical Sciences, Okayama, 700-8558 Japan; 2 Laboratory of Molecular Pharmacology, Division of Pharmaceutical Sciences, Kanazawa University Graduate School, Kanazawa, Ishikawa 920-1192 Japan; 3 Department of Molecular Biology and Biochemistry, Okayama University Graduate School of Medicine, Dentistry and Pharmaceutical Sciences, Okayama, 700-8558 Japan; 4 Laboratory for Animal Resource Development Unit and Genetic Engineering Team, RIKEN Center for Life Science Technologies, 2-2-3 Minatojima Minami, Chuou-ku, Kobe, Hyogo 650-0047 Japan

**Keywords:** Cell death in the nervous system, Neurodegeneration

## Abstract

The pathophysiological role of mammalian target of rapamycin complex 1 (mTORC1) in neurodegenerative diseases is established, but possible therapeutic targets responsible for its activation in neurons must be explored. Here we identified solute carrier family 38a member 1 (SNAT1, *Slc38a1*) as a positive regulator of mTORC1 in neurons. *Slc38a1*^*flox/flox*^ and *Synapsin I-Cre* mice were crossed to generate mutant mice in which *Slc38a1* was selectively deleted in neurons. Measurement of 2,3,5-triphenyltetrazolium chloride (TTC) or the MAP2-negative area in a mouse model of middle cerebral artery occlusion (MCAO) revealed that *Slc38a1* deficiency decreased infarct size. We found a transient increase in the phosphorylation of p70S6k1 (pp70S6k1) and a suppressive effect of rapamycin on infarct size in MCAO mice. Autophagy inhibitors completely mitigated the suppressive effect of SNAT1 deficiency on neuronal cell death under in vitro stroke culture conditions. These results demonstrate that SNAT1 promoted ischemic brain damage via mTOR-autophagy system.

## Abstract (web summary)

Yamada et al. demonstrate that solute carrier family 38a member 1 (SNAT1, Slc38a1) is as a positive regulator of mTORC1 that promotes neuronal cell death, using neuron-specific *Slc38a1* knockout mice. This study shows that SNAT1 exacerbates ischemic brain damage through mTOR-mediated suppression of autophagy.

## Introduction

Functional defects in neurons cause neurodegenerative diseases such as Alzheimer disease, amyotrophic lateral sclerosis, Parkinson disease, and cerebrovascular disease. These disorders are characterized by cellular damage and death in specific cerebral neurons, which are associated with physiological disabilities of patients, including tremor, paralysis, memory impairment, and cognitive dysfunction. Autophagy is a highly programmed self-degrading system, and its dysregulation occurs in neurodegenerative diseases^1^. The mammalian target of rapamycin (mTOR) complex 1 (mTORC1) suppresses the initiation of autophagy by phosphorylating ULK1, and mTOR activity is upregulated in Alzheimer disease, Parkinson disease, and Huntington disease^2,3^. Further, mTOR inhibition exerts therapeutic effects in models of neurodegenerative disease^3^, indicating the critical role of the mTOR-autophagy system in neuropathological disorders.

mTOR, which is an essential subunit of mTORC1, is a serine/threonine protein kinase of the PI3K family of protein kinases. By phosphorylating multiple substrates, mTORC1 regulates intracellular events including protein translation, glycolysis, lipid synthesis, and autophagy^4^. The small G protein RHEB, which resides on endosomal membranes, activates mTORC1, and the Tsc1/Tsc2 complex acts as a GTPase RHEB to inhibit mTORC1 activity. Inhibition of mTORC1 by Tsc1 in hippocampal neurons suppresses neuronal cell death by inducing autophagy in a model of ischemic stroke^5^. Further, the beneficial effects of inhibiting mTOR with rapamycin and its analogues are revealed by animal models of neurodegenerative disorders^6,7^. Although the pathophysiological role of mTORC1 in central nervous system is established, a therapeutic strategy that specifically targets mTORC1 in neurons is not available.

Among amino acids, l-glutamine (l-Gln) serves as a resource for energy production or protein synthesis and acts as an essential mediator of mTORC1 activation^8–11^. l-Gln is a substrate of solute carrier (Slc) family transporters including Slc1, Slc7, and Slc38s, and these transporters contribute to neurodegenerative diseases^12,13^. Although neurons express multiple Slc family members^12,14–17^, l-Gln transporters that are specifically expressed by neurons, which play a critical role in neuropathological diseases, have not been identified. Here we demonstrate that SNAT1, which is selectively expressed in neurons, acts as a positive regulator of mTORC1 in neurons and plays an important pathological role in the mechanism underlying neuronal survival after ischemia. Thus, our data provide insights into the therapeutic strategies for patients with neuropathological diseases.

## Results

### High expression of SNAT1 in neurons

**Fig. 1:**
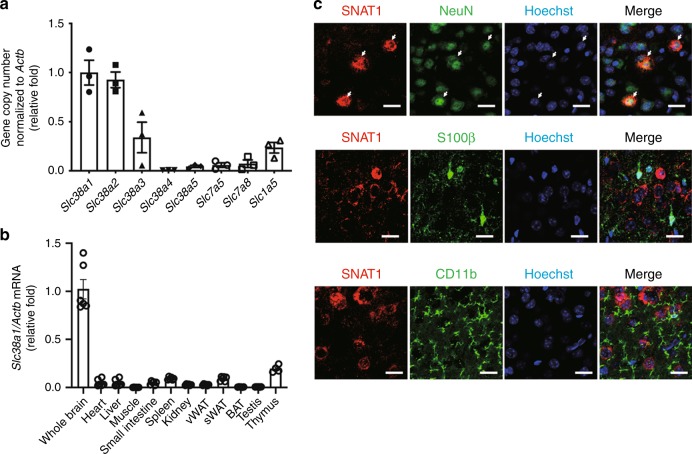
Analysis of *Slc38a1* expression in mouse tissues. **a** mRNA copy numbers of systems A (*Slc38a1*, *Slc38a2*, and *Slc38a4*), N (*Slc38a3* and *Slc38a5*), L (*Slc7a5* and *Slc7a8*), and ASC (*Slc1a5*) in the whole brain. Total RNA was extracted from whole brain, and the mRNA copy number of each gene was quantified using qRT-PCR. Values were normalized to those of *Actb* (*n* = 3). **b** Comparison of *Slc38a1* mRNA levels among mouse tissues. Total RNAs were extracted from the indicated tissues, and the mRNA levels of *Slc38a1* were compared using qRT-PCR. Values were normalized to those of *Actb*. (*n* = 4–6) **c** Identification of SNAT1-expressing cells in the cerebral cortex. Double-immunohistochemical using antibodies against SNAT1 and NeuN (neuron marker); single-immunohistochemical staining using antibodies against S100β (astrocyte marker), or CD11b (microglial marker). Nuclei were counterstained with Hoechst 33342. Scale bars = 20 µm. (vWAT, visceral white adipose tissue; sWAT, subcutaneous white adipose tissue; BAT; brown adipose tissue)

Certain members of the Slc38a family serve as net neutral amino acid transporters and use l-Gln as a preferred substrate. l-Gln indirectly activates mTORC1 via the simultaneous influx of essential amino acids (EAAs) and efflux of l-Gln, which are regulated by Slc7a5 or Slc7a8^11,18,19^. To identify Slc family transporters, which are characterized by a positive regulatory role in mTORC1 activity and highly specific expression in the brain, we first compared mRNA copy numbers of systems A (*Slc38a1*, *Slc38a2*, and *Slc38a4*), N (*Slc38a3* and *Slc38a5*), L (*Slc7a5* and *Slc7a8*), and ASC (*Slc1a5*) transporter genes. The levels of *Slc38a1* and *Slc38a2* were higher compared with those of other genes tested (Fig. 1a). When the mRNA levels of each Slc transporter were *Slc38a1* was predominantly expressed throughout the brain compared with other Slc family members (Fig. 1b and Supplementary Fig. 1a). Consistent with a previous report^20^, *SNAT1* mRNA and protein were expressed in brain segments including the cerebral cortex, hippocampus, striatum, hypothalamus, olfactory bulb, cerebellum, midbrain, and medulla-pons (Supplementary Fig. 1b, c). Immunohistochemical analysis of SNAT1 in the cerebral cortex revealed that SNAT1 was specifically expressed in NeuN-positive neurons but not in S100β-positive astrocytes or in CD11b-positive microglia (Fig. 1c). These results indicate that SNAT1 was preferentially expressed in neurons.

### Targeted deletion of *Slc38a1* from the genomes of neurons

**Fig. 2:**
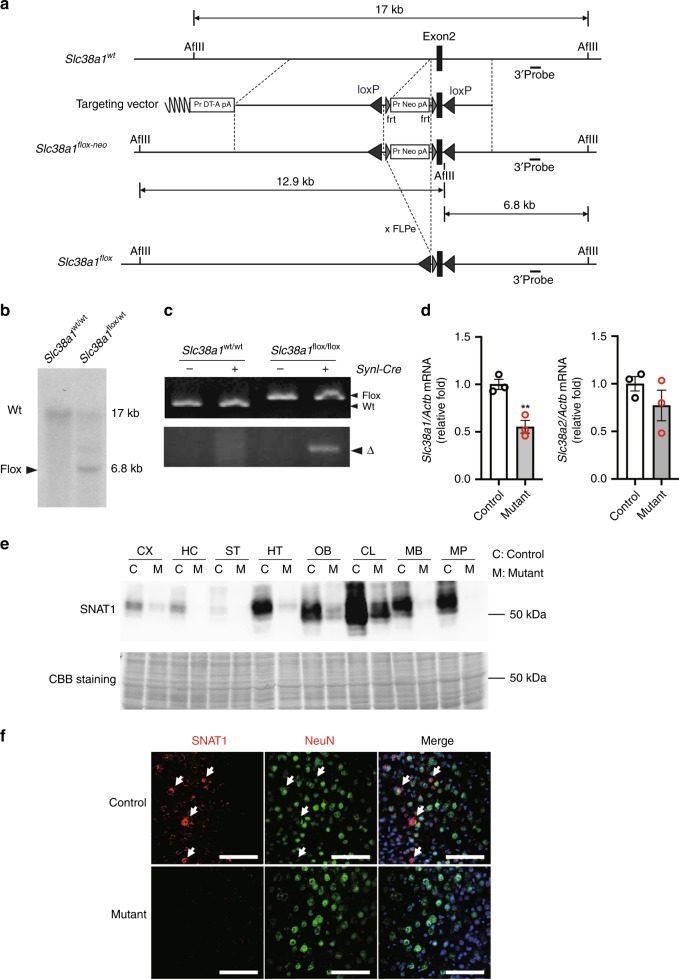
Generation of neuron-specific *Slc38a1* knockout mice. **a** Targeting strategy to create the floxed *Slc38a1* allele (*Slc38a1*^*flox*^). *Slc38a1* exon 2 is flanked by loxP sites. The flippase recombinase target-flanked Neo cassette was removed by crossing with CAG-FLP mice. Exon 2 was removed by crossing with *SynI-Cre* mice to selectively produce the Δ allele in neurons. **b** Southern blot analysis to confirm the recombination with the targeting vector at the genomic *Slc38a1* locus. Genomic DNA from embryonic stem cells was digested with AfIII and hybridized with a DIG-labeled 3′ probe. **c** PCR analysis verifying the Δ allele in mutant mice. Genomic DNA was extracted from the brain of each indicated mouse, and PCR products derived from the wild-type, flox, or Δ allele were detected. **d** Quantification of *Slc38a1* and *Slc38a2* mRNA levels in whole brains of from mutant mice. Total RNAs were extracted from whole brains of control or mutant mice, and the mRNA levels of *Slc38a1* and *Slc38a2* were compared using qRT-PCR. Values were normalized to those of *Gapdh* (*n* = 3). **e** Deletion efficiency of *Slc38a1* in brain segments. Proteins were extracted from each indicated brain segment of control or mutant mice, and SNAT1 was detected using western blotting. CBB staining was used as a loading control. C and M indicate control and mutant, respectively. **f** Confirmation of neuron-specific *Slc38a1* deficiency in mutant mice. Double-immunohistochemical staining using antibodies against SNAT1 and NeuN. Nuclei were counterstained with Hoechst 33342. Scale bars indicate 100 µm

We used Cre-loxP strategies to generate mutant mouse strain expressing a floxed allele of *Slc38a1* (Fig. 2a). The wild-type (WT) allele yielded a 17-kb fragment, whereas the homologous targeted mutant allele yielded a 6.8-kb fragment (Fig. 2b). To investigate the function of *Slc38a1* in neurons, the *Synapsin I (SynI)-Cre* system was employed to generate mutant mice in which *Slc38a1* could be selectively deleted from the genomes of SynI-positive neurons (Fig. 2a, b). Here, *Slc38a1*^*flox/flox*^ and *SynI-Cre*; *Slc38a1*^*flox/flox*^ mice are designated as control and mutant mice, respectively. The deleted allele was only detected in mutant mice (Fig. 2c). The level of *Slc38a1* mRNA was decreased, although that of *Slc38a2* was unchanged throughout the whole brain (Fig. 2d). The primer set used to detect *Slc38a1* mRNA recognizes exon 2 of *Slc38a1*, indicating that full-length *Slc38a1* mRNA might be expressed in the brain except by *SynI-Cre*-targeting cells of mutant mice. Consistent with this prediction, each brain segment isolated from mutant mice displayed decreased levels (<50%) of SNAT1 compared with those of controls (Fig. 2e). Further, SNAT1 expressing neurons were undetectable in the cerebral cortex of the mutants (Fig. 2f), indicate that our system specifically deleted *Slc38a1* from the genomes of neurons.

### Effect of neuron-specific *Slc38a1* deficiency on cerebral infarction

**Fig. 3:**
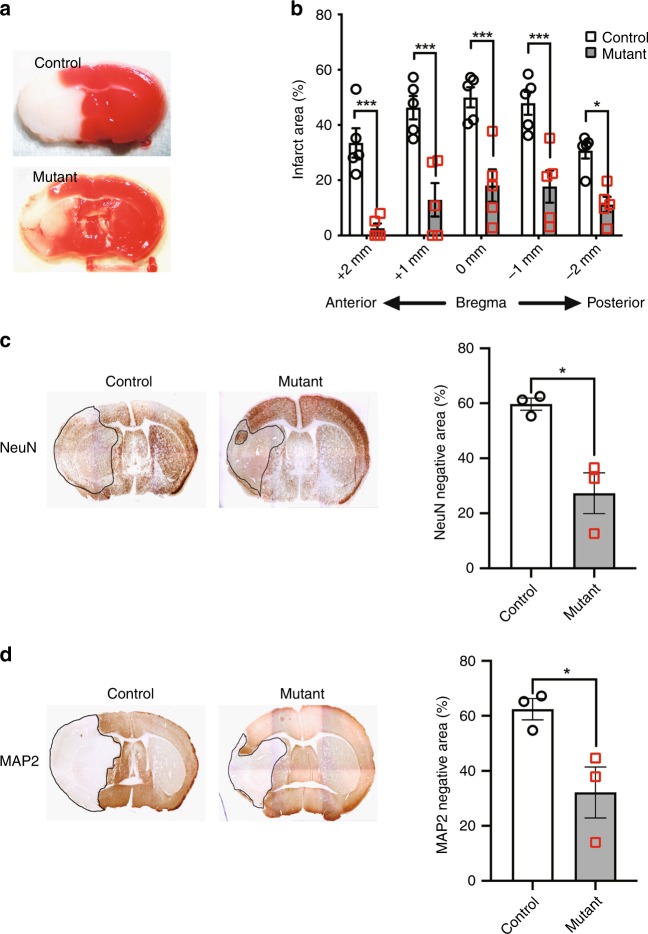
Neuron-specific *Slc38a1* deficiency confers resistance to ischemic brain injuries. **a**, **b** TTC staining of a brain section prepared from MCAO model mice. Representative images of the coronal bregma section **a** and measurements of the infarct area at each indicated point from the bregma (**b**, *n* = 5). **c**, **d** Immunohistochemical detection of the neurodegenerative effect of MCAO on cerebral neurons. Brain sections at the bregma were stained with antibodies against the neuronal markers NeuN (**c**, *n* = 3) or MAP2 (**d**, *n* = 3), and areas with undetectable staining were measured

We employed a model of the middle cerebral artery occlusion (MCAO) to simulate neurodegenerative disease and assessed ischemic brain injury in mutant mice. When the infarct area or volume was evaluated using immunohistochemical detection of TTC (Fig. 3a), mutant mice exhibited a smaller infarct area compared with that of the control (Fig. 3b). Further, immunohistochemical analysis revealed that the NeuN- or MAP2-negative area was smaller in mutant mice (Fig. 3c, d). These results demonstrate that SNAT1 expressed in neurons positively regulated ischemic brain injury.

### Critical role of mTORC1 activation via SNAT1 in ischemic brain injury

**Fig. 4:**
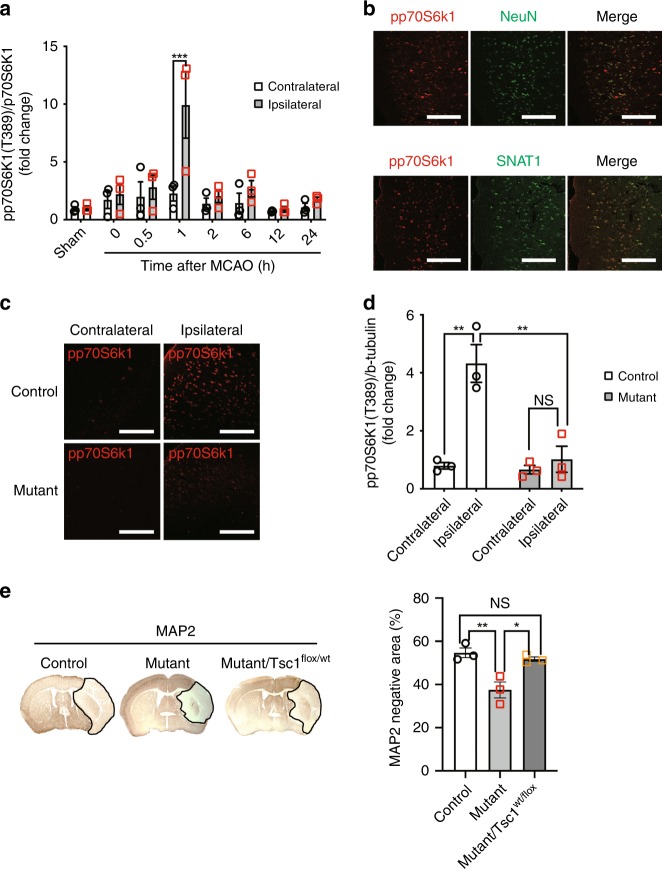
mTORC1 activation promotes ischemic brain injuries. **a** Assessment of mTORC1 activity in the brain after MCAO. Cerebral cortex samples were isolated from the contralateral or ipsilateral region at each indicated time point after MCAO, and the phosphorylation of p70S6k1 was compared by measuring the pp70S6k1(T389)/p70S6k1 ratio (*n* = 3). **b** mTORC1 activity in cerebral neurons after MCAO. Brain sections were prepared from mice 1 h after MCAO, and NeuN- or SNAT1 -positive cells in the cerebral cortex were double stained with an antibody against pp70S6k1(T389). Bars = 100 µm. **c**, **d** Inhibitory effect of neuron-specific *Slc38a1* deficiency on mTORC1 activation by MCAO. Whole brain samples were isolated from control or mutant mice 1 h after MCAO, and pp70S6k1(T389) levels in the cerebral cortex were compared using immunohistochemistry **c** or western blotting (**d**, *n* = 3). Bars = 100 µm. **e** Suppression of the neuroprotective effect of *Slc38a1* deficiency through *Tsc1* heterozygosity. Whole brain samples were prepared from control, mutant, or mutant/Tsc1^*flox/wt*^ mice after MCAO, and brain sections at the bregma were stained with an anti-MAP2 antibody to measure determine unstained areas (*n* = 3)

mTORC1 activation in neuropathological disorders such as ischemic brain injuries cause neuronal cell death^2,5^. p70S6K1 phosphorylates ribosomal protein S6 and regulates translation. p70S6K1 is directly phosphorylated at T389 by mTORC1, and its phosphorylation level serves as a surrogate for mTORC1 activity. When the pp70S6K1(T389)/p70S6K1 ratio was compared between the contralateral and ipsilateral regions 0.5, 1, 2, 6, 12, and 24 h after MCAO using western blotting, the phosphorylation level in the ipsilateral region increased 1 h after MCAO, followed by a decline to the same level detected in the contralateral region (Fig. 4a). Most NeuN- or SNAT1-positive cells expressed pp70S6K1(T389) in the ipsilateral region (Fig. 4b). To confirm the critical role of mTORC1 in ischemic damage, we mated mutant mice with *Tsc1* flox mice and generated mutant progeny harboring a heterozygous allele of *Tsc1*. Here, mice with such an allele were used, because mTORC1 hyperactivation in neuron associated with *Tsc1* or *Tsc2* deficiency induces behavioral defects and shortens survival^21,22^. Immunohistochemical and western blotting analyses revealed that the level of pp70S6K1(T389) in the ipsilateral region of mutant mice was lower compared with that of controls (Fig. 4c, d). Further, measurement of the MAP2-negative area revealed that Tsc1 heterozygosity completely counteracted the neuroprotective effect of *Slc38a1* deficiency (Fig. 4e). Consistent with previous reports^23–25^, rapamycin administration also decreased infarction area (Supplementary Fig. 2). These results demonstrate that mTORC1 activation via SNAT1 caused ischemic brain injury.

### Effect of *Slc38a1* deficiency on mTORC1 activity

**Fig. 5:**
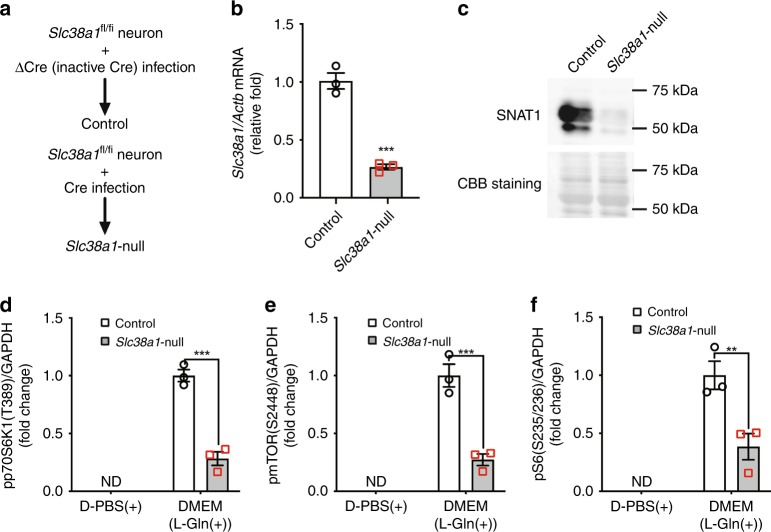
SNAT1 activates mTORC1 in cerebral neurons. **a** Procedure for deleting *Slc38a1* using an in vitro culture system. Primary neurons isolated from the cerebral cortices of Slc38a1^*flox/flox*^ mice were infected with a lentivirus encoding inactive ΔCre or active Cre, and control or Slc38a1-null neurons were prepared. **b**, **c** Deletion efficiency of *Slc38a1* from primary neurons. Total RNA and protein were extracted from control or *Slc38a1*-null neurons, and the expression levels of Slc38a1 were assessed using qRT-PCR (**b**, *n* = 3) or western blotting (**c**, *n* = 3). **d**–**f** Analysis of mTORC1 activity after *Slc38a1* deficiency. Proteins were extracted from control or *Slc38a1*-null neurons, and pp70S6k1(T389), pmTOR(S2448), and pS6(S235/236) were detected using western blotting. GAPDH served as a loading control (*n* = 3)

To test the possibility that l-Gln incorporated via SNAT1 promotes mTORC1 activity, primary neurons were isolated from *Slc38a1*^*flox/flox*^ mice, and Cre recombinase (Cre) or ΔCre (inactive form of cre recombinase) was introduced using a lentiviral vector. Here, Cre- and ΔCre-introduced neurons were designated *Slc38a1*-null and control cells, respectively (Fig. 5a). Compared with control neurons, *Slc38a1*-null neurons expressed decreased levels of both mRNA and protein (Fig. 5b, c). Further, the rate of L-Gln incorporation into Slc38a1-null neurons was slower compared with that of the control (Supplementary Fig. 3a). To assess the effect of *Slc38a1* deficiency on the phosphorylation of mTORC1-target molecules including p70S6K1, mTOR, and S6 cells were cultured in PBS for 3 h and then stimulated with PBS or DMEM (l-Gln[+]) for 5 h. The level of pp70S6K1(T389), pmTOR(S2448), and pS6(S235/236) was lower in *Slc38a1*-null neurons (Fig. 5d–f). Further, 1 h treatment with l-Gln transporter inhibitors such as MeAIB and 2-aminobicyclo-(2,2,1)-heptane-2-carboxylic acid (BCH) decreased the levels of pp70S6K1(T389), pmTOR(S2448), and pS6(S235/236) in primary neurons (Supplementary Fig. 3b–d). The addition of l-Gln and EAAs to PBS increased the level of pp70S6K1(T389) in the mouse neuroblastoma cell line Neuro2a, indicating that l-Gln and EAAs were required for full mTORC1 activation (Supplementary Fig. 3e). These results indicate that SNAT1 positively regulated mTORC1 activity.

### Effect of *Slc38a1* deficiency on autophagy in primary neurons

**Fig. 6:**
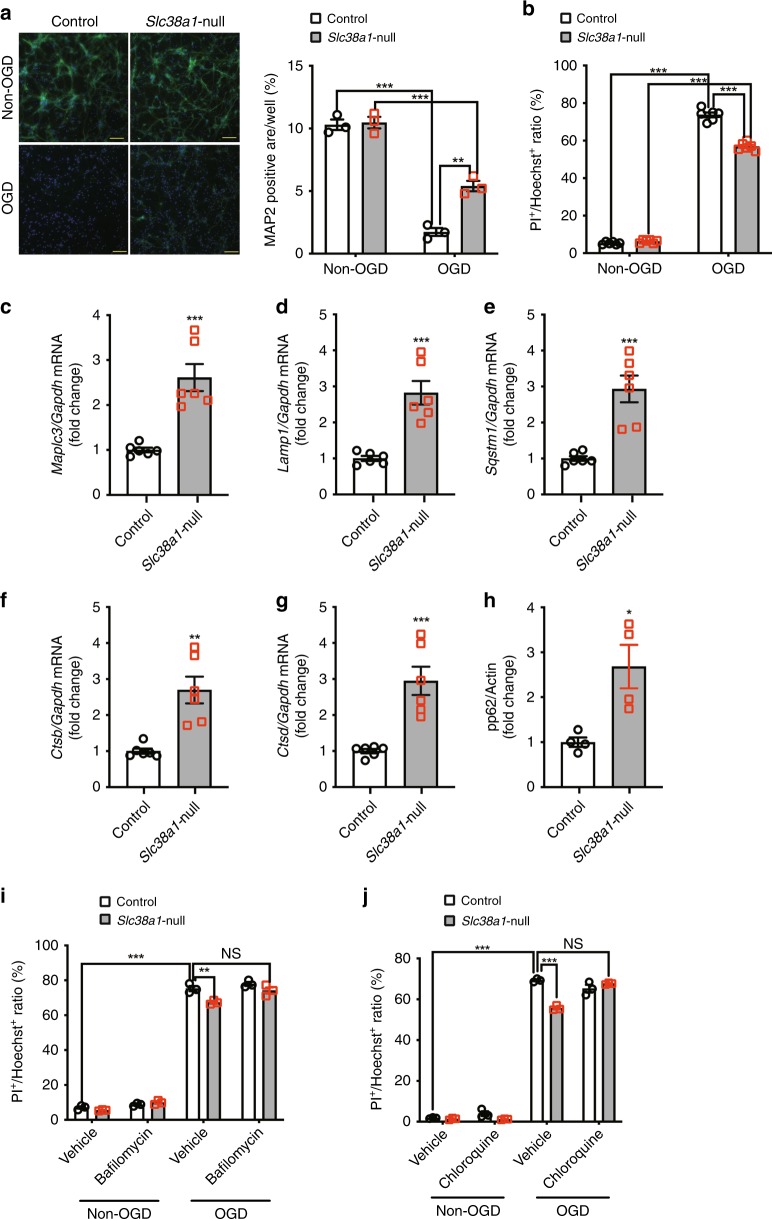
Autophagy is a critical mediator of neuroprotection conferred by *Slc38a1* deficiency. **a**, **b** Assessment of the neuroprotective effect against ischemic stress conferred by *Slc38a1* deficiency. Control or *Slc38a1*-null neurons were cultured under normal (anaerobic glucose deprivation [OGD]) or OGD conditions and stained with an anti-MAP2 antibody (a, *n* = 3) or PI (b, *n* = 6) to evaluate dead neurons. Bars = 100 µm. **c**–**g** Increases in the mRNA levels of autophagy-related genes associated with *Slc38a1* deficiency. Total RNA was extracted from control or *Slc38a1*-null neurons, and the mRNA levels of each indicated gene were measured using qRT-PCR. Values were normalized to those of *Gapdh* (*n* = 6). **h** Evaluation of autophagy under ischemia. Cell-free lysates of Slc38a1-null neurons cultured under OGD were subjected to western blotting using anti-phospho-p62 and anti-actin antibodies (n = 4). **i**, **j** Suppressive effect of autophagy inhibitors on neuroprotection induced by *Slc38a1* deficiency. Control or *Slc38a1*-null neurons were treated with 1 nM bafilomycin **i** or 5 µM chloroquine **j** in the presence or absence of OGD. Neuronal cell death was assessed using PI staining (*n* = 3)

Autophagy, which is inhibited by mTORC1, regulates neuroprotection^1,5,26^. We assessed the contribution of autophagy to the neuroprotective effect of Slc38a1 deficiency. Oxygen-glucose deprivation (OGD) serves as an in vitro model of stroke, and reflects in vivo models of ischemic stroke. Although there was no difference in the MAP2-positive area under normal culture conditions, *Slc38a1*-null neurons became resistant to OGD, because the MAP2-positive area was larger compared with that of the control (Fig. 6a). When cell death was assessed using PI staining, there were fewer PI^+^ cells in the *Slc38a1*-null group under the OGD (Fig. 6b). Similar results were obtained when primary neurons or Neuro2a cells were treated with MeAIB under OGD (Supplementary Fig. 4a, b). qRT-PCR analysis of autophagy-regulatory genes revealed that *Slc38a1*-null neurons displayed increases in the levels of *Map1lc3b*, *Lamp*, *Sqstm1*, *Ctsb*, and *Ctsd* mRNAs compared with those of the controls (Fig. 6c–g). Phosphorylation of p62 is specific to autophagy. *Slc38a1*-null neurons expressed higher levels of phosphorylated p62 compared with those of control neurons under OGD (Fig. 6h). Further, autophagy inhibitors such as bafilomycin and chloroquine decreased the inhibitory effect of *Slc38a1* deficiency on OGD-induced cell death (Fig. 6I, j). Consistent with this observation, the suppressive effect of MeAIB on OGD-induced cell death was abolished by chloroquine (Supplementary Fig. 4c). These results demonstrate that SNAT1 promoted neuronal cell death by inhibiting autophagy.

## Discussion

Here we show the neurotoxic effect of SNAT1 expressed by cerebral neurons in a model of ischemic infarction. Emerging evidence reveals that SLC transporters regulate the intracellular levels of nutrients and drugs and play critical roles in disease^12^. Among SNAT family transporters, SNAT1 are upregulated in solid tumors such as hepatocellular carcinoma, breast cancer and osteosarcoma^27–29^. Jin et al. used a Rett syndrome model to show that upregulation of SNAT1 expression in microglia contributes to NMDA receptor-dependent neurotoxicity^30^. These results indicate that SNAT1 is associated with the pathogenesis of certain diseases. Here we found that SNAT1 was predominantly expressed in cerebral neurons (Fig. 1d), and *Slc38a1* deficiency did not conferred any behavioral disorders (Supplementary Fig. 5) but resistance to ischemic brain damage (Figs 3, 6). Although several types of neurons express SNAT1, including cholinergic motor neurons, dopaminergic neurons in the substantia nigra, and GABAnergic interneurons^17,20^, we suggest that SNAT1-positive cortical neurons were primarily affected during ischemic damage, because the infarction areas of cortical region were reduced by *Slc38a1* deficiency (Fig. 3c). The detailed functions of SLC transporters, particularly those in the central nervous system, during ischemic damage should be analyzed to better understand their pathological roles or potential as therapeutic targets.

mTORC1 is aberrantly activated in tumors^31^ and it can induce malignant phenotypes such as increased proliferative capacity and cancer stem-like properties^32–34^. SNAT1 influences the malignant phenotype through activation of mTORC1^29,35,36^. However, the functional relationship between mTORC1 and SNAT1 in neurons of the central nervous system or in ischemic brain damage is unknown. To our knowledge, this present study is the first to report a neurotoxic effect of mTORC1 activated by SNAT1 under ischemia. The ipsilateral region of MCAO model mice exhibited a transient increase in mTORC1 activity (Fig. 4a), and the neuroprotective effect of *Slc38a1* deficiency was abolished by Tsc1 heterozygosity (Fig. 4e), indicating the SNAT1 promoted ischemic infarction by activating mTORC1. Consistent with our present results, Tsc1 inhibits mTORC1 and induces a neuroprotective effect on hippocampal neurons^5^, suggesting that hyperactivation of mTORC1 is toxic to several types of neurons.

The mechanism through which mTORC1 regulates neuronal cell death may not be easy to determine, because systemic administration of rapamycin, an mTORC1 inhibitor, has neuroprotective effects on ischemic brain damage^37–39^. However, in contrast to the results of the present study the mTORC1 activity of all cells in various tissues, including the brain, should have been inhibited in the studies cited, indicating that mTORC1 in glial cells, microglia, or other cells confer neuroprotection. SNAT1 was specifically expressed in neurons but not in glial cells or microglia (Fig. 1c) Other mechanisms may therefore induce neuroprotection by activating mTORC1 in cells exposed to ischemic stresses. Future studies are required to determine how the mTORC1 activity of each cell in brain tissues contributes to the pathogenesis of ischemic infarction or to brain damage. Further, full activation of mTORC1 requires L-Gln as well as EAAs such as leucine (Supplementary Fig. 3). Further, we found that MeAIB decreased, but could not completely prevent cell death caused by OGD (Supplementary Fig. 4), suggesting that system L transporters mediate OGD-induced phenotypes.

Autophagy is a highly conserved self-degrading system that mediates stress responses. Recent studies reveal the function of autophagy in cell death, aging, tumorigenesis, metastasis, and drug resistance^40–44^. Moreover, autophagy contributes to neuroprotection against ischemic damage^5,45–50^. Autophagy is negatively regulated by mTORC1-mediated phosphorylation of ATG1 or ATG13^4^, and its activation via inhibition of mTORC1 is associated with neuroprotective effects^5,25^. Here we show that *Slc38a1* deficiency decreased mTORC1 activity (Fig. 5d–f) and increased the expression levels of genes associated with autophagy (Fig. 6c–g). Further, inhibition of autophagy with bafilomycin or chloroquine decreased the suppressive effect of *Slc38a1* deficiency on OGD-induced neuronal cell death (Fig. 6i, j). These results are consistent with those of a previous report demonstrating the inhibitory effect of bafilomycin on neuroprotection induced by autophagy in a model of cerebral ischemic^51^. Activation of autophagy induced by mTORC1 inhibition or TFEB overexpression in neurons promotes neuroprotection in models of cerebral ischemic^5,52^. Therefore, the neuron-selective activation of autophagy or inhibition of mTORC1 may prevent ischemic brain damage. Therefore, SNAT1 may serve as a potential therapeutic target because its expression is largely restricted to brain or central neurons (Fig. 1b, c). Although only in vitro assays were performed here to test the neuroprotective effect of autophagy activated by *Slc38a1* deficiency, our data and those of previous reports support the hypothesis that administration of inhibitors of autophagy such as bafilomycin or chloroquine will inhibit the neuroprotective effects conferred by *Slc38a1*-deficiency during cerebral ischemic damage.

In conclusion, our study demonstrates that SNAT1 shows promise as a therapeutic target to prevent neuronal cell death caused by ischemic stress. We expect that our findings will provide insight into the neuropathological interactions between members of the SLC transporter family and other signaling pathways.

## Methods

### Real-time–quantitative RT-PCR (qPCR)

Total RNA was extracted from cells or tissues, followed by cDNA synthesis using reverse transcriptase and oligo-dT primers. The cDNA samples were then used as templates for real-time PCR analysis using gene-specific primers (Supplementary Table 1). qPCR was performed using a MiniOpticon real-time PCR system (BioRad). The cycle parameters were as follows: denaturation at 95 °C for 30 s, annealing for 30 s at 62 °C, and elongation for 30 s at 72 °C. The expression level of each gene was calculated using the ΔΔCt method or quantification of copy number.

### Immunohistochemistry

Mice were deeply anesthetized via intracardial perfusion with PBS and 4% paraformaldehyde. Brains were removed, fixed overnight, and cryoprotected in 30% sucrose. Free-floating sections (30-µm thick) were prepared using a cryostat and stored at −20 °C in cryoprotective solution (30% sucrose, 30% ethylenglycol, and 1% polyvinylpyrrolidone in PBS). Sections were washed in Tris-buffered saline containing 0.1% Tween 20 (TBST) and incubated for 15 min in TBST. After washing in TBST, sections were incubated for 1 h in blocking solution (2% normal goat serum in TBST), followed by incubation at 4 °C overnight in blocking solution containing a primary antibody against SNAT1 (kindly provided by Jeffrey D. Erickson; 1:200), NeuN (MAB377 Chemicon; 1:400), S100β (S2532 Sigma-Aldrich; 1:400), CD11b (MCA711G Serotec; 1:200) or phospho-p70 S6 kinase (Thr389) (#9234 Cell Signaling Technology; 1:200). After rinsing in TBST, sections were incubated for 2 h at room temperature with blocking solution containing an anti-rabbit IgG antibody conjugated to Alexa 594 (Invitrogen; 1:400), an anti-mouse IgG antibody conjugated with Alexa 488 (Invitrogen; 1:400), or an anti-rat IgG antibody conjugated with Alexa 488 (Invitrogen; 1:400), rinsed in TBST and incubated for 15 min in TBST containing Hoechst 33342 at room temperature. After washing in TBST, sections were mounted onto slides in FluorSave (Calbiochem). Confocal images were acquired using an LSM710 microscope and ZEN2009 software (Zeiss).

### Generation of mutant mice

Three splice variants of Slc38a1, revealed by a search of the Ensemble database (http://www.ensembl.org/index.html) contain exon 2 (chromosome 15: 96,624,169–96,623,956, ENSMUSE00000621499), which encodes the start codon for the SLC38a1. Therefore, we generated conditional knockout mice with a floxed exon 2 of the *Slc38a1* locus (Accession No. CDB0835K)^58^. The targeting vectors harboring loxP sites and a neomycin resistant (Neo^R^) cassette were used to electroporate TT2 embryonic stem (ES) cells^53^. DNAs were extracted from different ES cell clones after neomycin selection for the mutant allele, followed by Southern blotting using a [^32^P]-labeled probe specific for the target region of genomic DNA digested with AflII. The WT and the corresponding mutant allele yielded 17-kb 6.8-kb fragments, respectively. ES cells containing the floxed allele were injected into CD-1 8-cell stage embryos to generate chimeric mice. To remove the Neo^R^ cassette flanked by flippase recombinase target sequences, offspring were crossed with flippase transgenic mice. *Slc38a1*^*flox/+*^ mice were crossed with *SynapsinI-Cre*^54^ to generate *SynapsinI-Cre*;*Slc38a1*^*flox/+*^ mice, and their progeny were intercrossed to obtain *SynapsinI-Cre;Slc38a1*^*flox/flox*^ mice. Tsc1^fl/fl^ mice were obtained from the Jackson Laboratory (stock #005680)^55^, and genotyping was performed using PCR to amplify genomic DNA isolated from the tail. The presence of the 3′ loxP site was verified using PCR with primers (Supplementary Table 2). The Committees on Animal Experimentation of Kanazawa and Okayama Universities approved the experiments using mice. The study was performed in compliance with the University’s Guidelines for the Care and Use of Laboratory Animals.

### Immunoblotting

Tissues or cultured cells were solubilized in lysis buffer containing 1% Triton X-100. Samples were subjected to SDS-PAGE, followed by electrophoretic transfer to polyvinylidene fluoride membranes and subsequent immunoblotting. Immunoblotting was performed using antibodies targeting SNAT1 (kindly provided by Jeffrey D. Erickson), phospho-p70 S6 kinase (Thr389) (#9234 Cell Signaling Technology), p70 S6 kinase (#2708 Cell Signaling Technology), phospho-mTOR (Ser2448) (#2971 Cell Signaling Technology), phospho-S6 (Ser235/236) (#4858 Cell Signaling Technology), phospho-Akt (Ser473) (#4060 Cell Signaling Technology), GAPDH (sc-25778 Santa Cruz Biotechnology, Inc.), β-tubulin (T4026 Sigma-Aldrich), and β-actin (sc-4778 Santa Cruz Biotechnology, Inc.). Primary antibodies were diluted 2000-fold with blocking solution (5% skim milk in TBST [137 mM NaCl, 0.05% Tween 20, 20 mM Tris–HCl buffer, pH 7.5]).

### MCAO procedure

MCAO surgery was performed according to previously published procedures^56^. Briefly, adult male mice were anesthetized with 35 mg/kg (i.p.) pentobarbital, and they recovered from anesthesia after approximately 30 min. The left common carotid artery was exposed through a mid-neck incision and dissected free from surrounding nerves for clipping. The occipital artery branches of the external carotid artery were isolated and then ligated. The internal carotid artery was isolated and separated from the adjacent vagus nerve, and the pterygopalatine artery was clipped. A 6-cm 3–0 monofilament nylon suture was inserted via the proximal external carotid artery into the internal carotid artery and then into the circle of Willis to effectively occlude the MCA. Animals were subjected to MCAO for 2 h and then killed to compare infarct volumes after reperfusion of blood flow for 24 h. To maintain the rectal temperature at 37 °C ± 0.5 °C, mice were placed onto a homeothermic blanket throughout surgery (Harvard Apparatus, Holliston, MA, USA.). For analgesia during MCAO procedures, 7 mg/kg lidocaine hydrochloride (Wako) was subcutaneously injected before surgery. Neurobehavioral assessments, including posture and rotation tests (blinded), were performed after the recovery time of 0 or 24 h. Actually, we used all mice that experienced MCAO surgery and found all of them had some defects in both tests (Supplementary Fig. 2 and Supplementary Fig. 6), indicating that no mice were excluded in this study. Brain samples for TTC stain and IHC analysis were collected at 24 h and 4 days after MCAO surgery, respectively. For staining using an anti-MAP2 antibody, fixed brains were embedded in paraffin and sectioned (5-μm thick). Sections were incubated with an anti-MAP2 antibody (M4403, Sigma-Aldrich), and immunostaining was conducted using the VECTASTAIN ABC kit (Vector Laboratories, Burlingame, CA, USA).

### Primary neuronal culture

The protocol employed in this study met the guidelines of the Japanese Society for Pharmacology and was approved by the Committee for the Ethical Use of Experimental Animals of Kanazawa University. All efforts were made to minimize animal suffering, reduce the number of animals, and utilize alternatives to in vivo techniques. Primary cultures of cortical neuronal were obtained from 15-day embryonic mice. Briefly, the cerebral cortex was dissected and incubated with 0.25% trypsin at 37 °C for 20 min. Cells were treated with serum and 0.1 mg/ml DNase and then mechanically dissociated using a sterile pipette tip. Dissociated cells were plated at 400,000 cells/cm^2^ on plastic dishes coated with 7.5 μg/ml poly-l-lysine, after we performed a trypan blue dye exclusion test. The cells were maintained in Neurobasal medium (Invitrogen) supplemented with 2% B27 supplement (Invitrogen) for 8 days at 37 °C in a humidified atmosphere containing 5% CO_2_, with medium replacement every 4 days. Neurons were activated with 5 mM glutamine (sigma) or ×100 essential amino acid (EAA) solution (Thermo Fisher).

### Lentivirus stocks for infecting primary cultures of neurons

Lentiviral vectors expressing Cre recombinase or ΔCre (inactive form) were kindly donated by Zhiping P. Pang (Robert Wood Johnson Medical School, NJ, USA). Three packaging expression plasmids (pRSV-REV, pMDLg/pRRE, and a vesicular stomatitis virus G [VSVG]) were kindly provided by Dr. Sudhof (Stanford University, Palo Alto, CA, USA). The lentivirus expression vector, pRSV-REV, pMDLg/pRRE, and the VSVG expression expressing plasmid were used to cotransfect HEK293T cells (RIKEN, Saitama, Japan) at 10, 2.5, 5, and 3 μg of DNA per 56.7-cm^2^ culture area, respectively, using the calcium phosphate method. After transfection (24 h), the HEK293T culture medium was replaced with DMEM, followed by culture for 48 h and subsequent collection of the culture medium. The culture medium was centrifuged at 500 × *g* for 5 min, and the supernatant containing lentivirus particles was directly added to the culture medium. Cre or ΔCre was fused to GFP, and the lentiviral titer could therefore be determined by measuring the fluourescence emitted by EGFP. The lentiviral supernatant was added at a ratio = 1:3 of lentiviral supernatant, yielding an MOI = 10. Primary cultures of neurons infected for 12 h on day 4 and analyzed on day 8. All experiments were performed using biosafety level II conditions.

### Oxygen glucose deprivation (OGD)

We induced OGD condition using an AnearoPack (Mitubishi Gas Chemical) and 50 mM 2-deoxy-glucose. Neurons were cultured for 24 h under OGD, fixed with 4% paraformaldehyde, and then reacted with anti-MAP2 antibody (M4403 Sigma-Aldrich)/Alexa Fluor 488-conjugated goat anti-mouse IgG (1:400, Invitrogen) for immunodetection. Cells were observed using a fluorescence microscope (BZ-8100; Keyence, Osaka, Japan). MAP2-positive areas were measured in four different visual fields per well, which were randomly chosen in a blinded fashion. The dead cells were analyzed using PI (10 μM) and Hoechst 33342 (10 μM) for 10 min. Stained cells were analyzed using an In Cell Analyzer 2000 (GE Healthcare). The cell death was calculated according to the ratio of PI-positive cells to Hoechst 33342-positive cells.

### Data analysis

Data analysis was performed using Prism 7, and the data are shown as the mean ± S.E. Statistical significance was determined using a two-tailed *t* test and unpaired one-way or two-way ANOVA with the Tukey posthoc test. *p < 0.05, **p < 0.01, ***p < 0.001.

### In situ hybridization

Brains were removed, fixed overnight, and cryoprotected in 30% sucrose. Brains were then dissected to prepare frozen sections (10-μm thick) using a cryostat (Leica CM 3050), and in situ hybridization was performed, as previously described^57^ using DIG-labeled cRNA probes. DIG-labeled cRNA probes for *Slc38a1* were prepared in vitro using T7 or SP6 RNA polymerase to transcribe *Slc38a1* cDNA fragments (919 bp) subcloned into pGEMT-Easy vectors (Promega). An *Slc38a1* cDNA fragment was obtained from a library of mouse brain cDNAs using the forward primer 5′-TCTGACTTCGGTGACACTGC-3′ and reverse primer 5′-CTTCATGGAGGGGATGAAGA-3′.

### Culture of Neuro2a cells

Neuro2a cells derived from a mouse neuroblastoma exhibited the neuronal stem cell-like ability to differentiate into neuron-like cells in the presence of all-trans retinoic acid (ATRA). Undifferentiated Neuro2a cells were cultured in DMEM supplemented with 10% FBS and passaged at least three times before treatment with ATRA. Neuro2a cells (25,000 cells/cm^2^) were then plated in DMEM supplemented with 10% FBS for 24 h, followed by a medium change to DMEM supplemented with 2% FBS and 20 μM ATRA to induce differentiation. Cultures were maintained at 37 °C for 2 days in a humidified atmosphere containing 5% CO_2_.

### L-Gln incorporation assay

In total 4 × 10^5^ primary neurons were suspended in Neurobasal medium containing B27 and seeded into the wells of a 24-well plate. After culturing for 4 days, neurons were incubated with lentivirus particles for 12 h. Culture media were replaced with fresh Neurobasal medium containing B27, and the L-Gln incorporation assay was performed after 4 days. Briefly, neurons were incubated in HEPES-buffered Krebs-Ringer solution (HKR) with 1.8 mM CaCl_2_ and 5.5 mM Glucose for 30 min. Neurons were then treated with 10 µM [^3^H] L-Gln for ≤ 15 min at 37 °C. Reactions were stopped by washing with HKR containing 5 mM cold L-Gln. Cell lysates were prepared by incubating them with 0.1 N NaOH for 1 h and the neutralized with 0.1 N HCl. The radioactivities of each cell lysate were measured using a liquid scintillation counter.

### Behavior analysis

The three-chamber tests for sociability were performed using a rectangular, three-chambered box with two dividing walls allowing access into each chamber. Mice were first placed in the middle chamber and allowed to explore for 10 min with free access to all parts of arena. After habituation, an unfamiliar mouse (naïve C57BL/6 male) was placed in the wire cage (in the right chamber); another wire cage (in the left chamber) was empty, and the test mouse was placed in the center compartment of the social test box and allowed to explore for a 10 min session, with free access into the two side chambers. The behavior of the animals was videotaped, tracked and then analyzed with the spent time in each chamber^59^.

The elevated plus maze was constructed with 2 open- and 2 closed-plastic arms. Mice were placed in the center of an elevated plus maze and were allowed to explore the maze for 10 min. Experiments were videotaped and then scored with the time spent in the closed arms (4 paws within closed arm) or open arms (4 paws within open arms).

For hole board test, mice were placed on an arena with regularly four arranged holes on the floor. The frequency of spontaneous elicited hole-poking behavior are measured during 5 min.

### Reporting summary

Further information on research design is available in the Nature Research Reporting Summary linked to this article.

## Supplementary information

Supplementary Information

Description of Additional Supplementary Files

Supplementary Data 1

Reporting Summary

## Data Availability

All data generated during and/or analyzed during this study are included in this published article and its supplementary information. The source data underlying graphs shown in main figures are presented in Supplementary Data 1. Full blots are shown in Supplementary Information.
