# Supplementary material for: Inhibition of the glutamine transporter SNAT1 confers neuroprotection in mice by modulating the mTOR-autophagy system
Source: Commun Biol. 2019 Sep 18;2:346. doi: 10.1038/s42003-019-0582-4 (PMC6751179; doi:10.1038/s42003-019-0582-4)
Supplement: Supplementary file 1 — Supplementary Information [file 42003_2019_582_MOESM1_ESM.pdf]

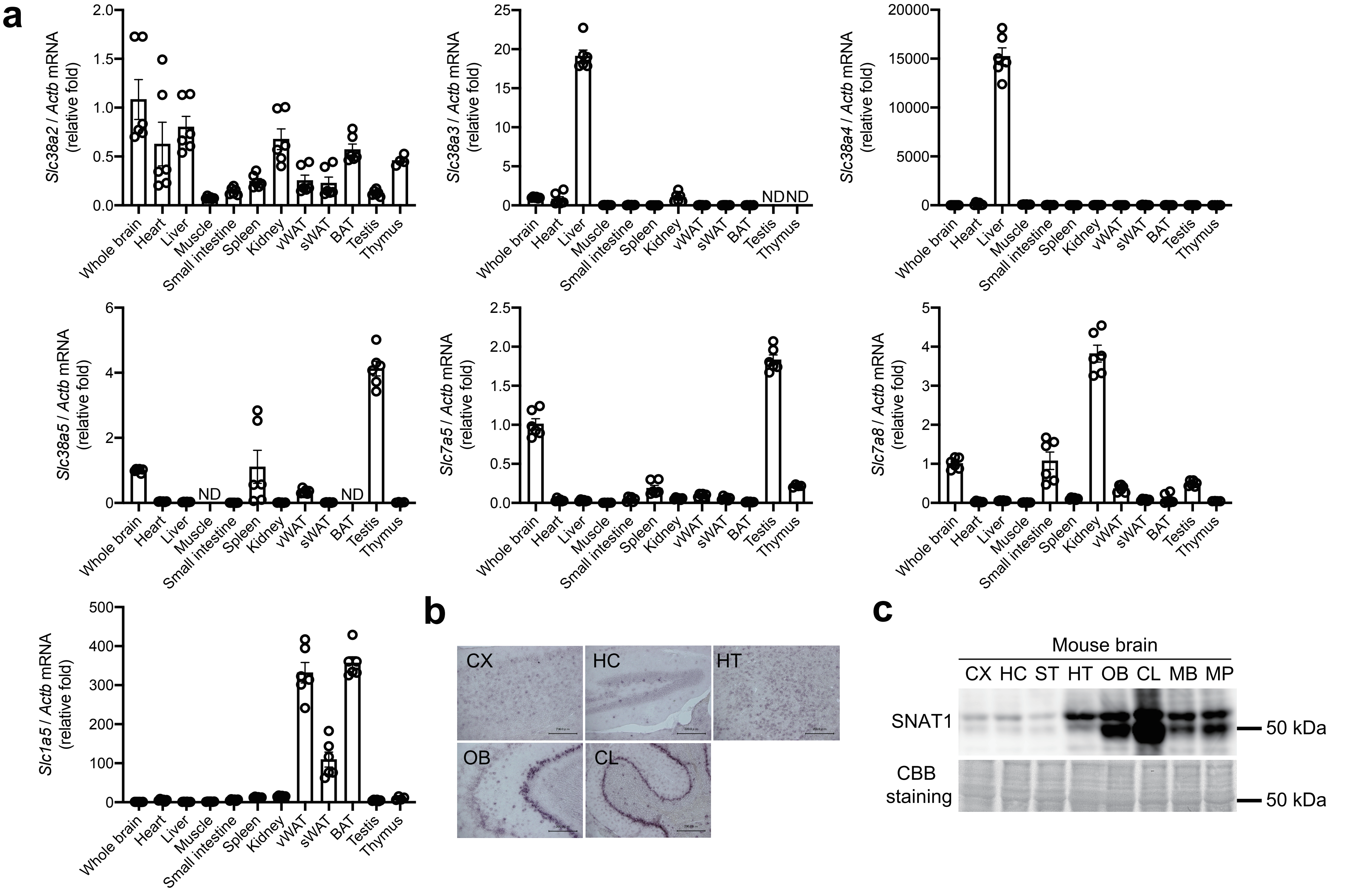

Supplementary Figure 1. Expression pattern of solute carrier family 38a members in the brain.
(a) Comparison of systems A (*Slc38a2, and Slc38a4*), N (*Slc38a3 and Slc38a5*), L (*Slc7a5 and Slc7a8*), and ASC (*Slc1a5*) transporter mRNA levels among mouse tissues (n = 4 - 6). (b) In situ hybridization assay of the whole brain using an *Slc38a1*-specific probe. (c) Expression profile of SNAT1 in mouse brain segments. After extracting proteins from each indicated brain segment, SNAT1 was detected using western blot analysis. CBB staining was used as a loading control. (CX, cortex; HC, hippocampus; ST, striatum; HT, hypothalamus; OB, olfactory bulb; CL, cerebellum; MB, midbrain; MP, medulla-pons.)

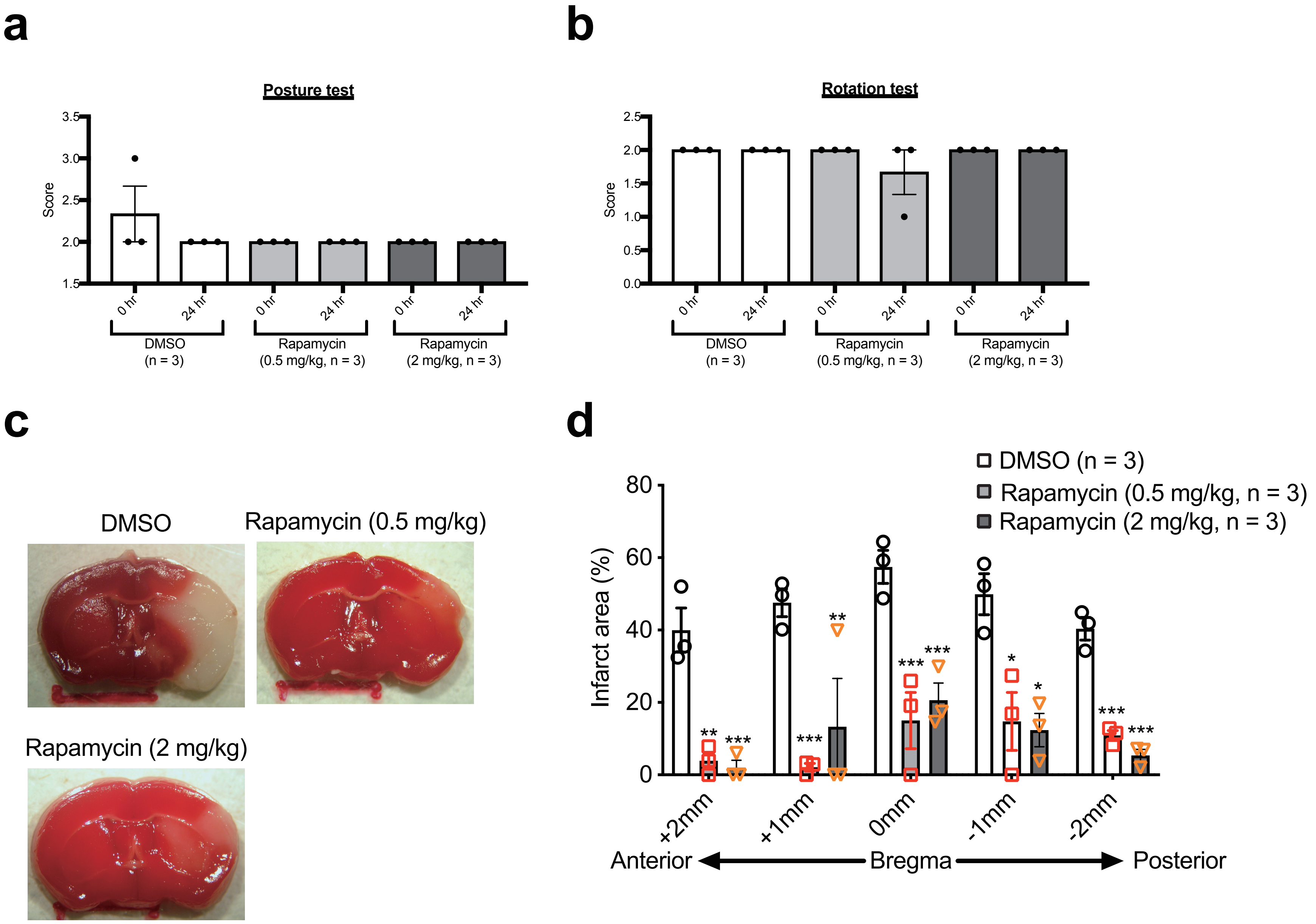

Supplementary Figure 2. Neuroprotective effect of rapamycin on ischemic infarction

(a, b) Exclusion criteria. Neurological symptoms were assessed 24 hours after surgery by posture or rotation test. Mice that had more than score 1 in either test were used for further experiments. (c, d) TTC staining of a brain section prepared from MCAO model mice. After the surgery, DMSO or each concentration of rapamycin was administered intraperitoneally and brain samples were collected at 24 hr after administration. Representative images of the coronal bregma section (c) and measurements of the infarct area at each indicated point from the bregma (d) were shown.

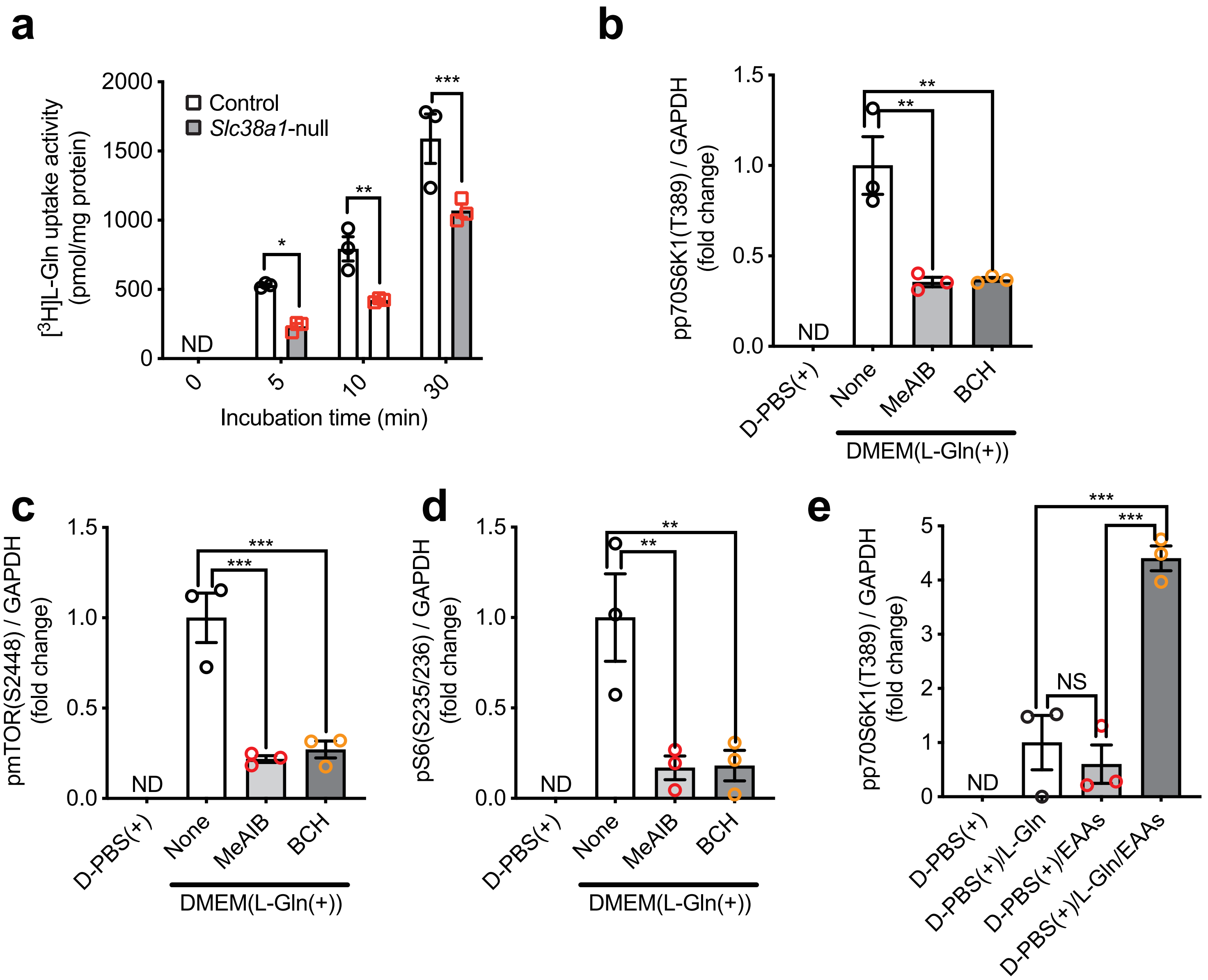

Supplementary Figure 3. L-glutamine (L-Gln) and essential amino acids (EAAs) are required for full activation of mTORC1.
(a) L-Gln incorporation assay. After the isolation of primary neurons from Slc38a1fl/fl mice, ΔCre or Cre lentivirus was infected and the radioactivity of 3H L-Gln at each indicated time point was measured. (b-d) Analysis of mTORC1 activity after L-Gln transporter inhibition. Primary neurons were treated with 20 mM methylaminoisobutyric acid (MeAIB) or 20 mM 2-aminobicyclo-(2,2,1)-heptane-2-car-boxylic acid (BCH) for 2 h. Proteins were extracted to compare the expression levels of pp70S6k1(T389) (b, n = 3), pmTOR(S2448) (c, n = 3), and pS6(S235/236) (d, n = 3) using western blotting. GAPDH served as a loading control. (e) Activation of mTORC1 by L-Gln and EAAs. After 3 h of culture in PBS, Neuro2a cells were stimulated with L-Gln, EAAs, or L-Gl-n/EAAs for 30 min. Proteins were extracted to compare the expression level of pp70S6k1(T389). GAPDH served as a loading control (n =3)

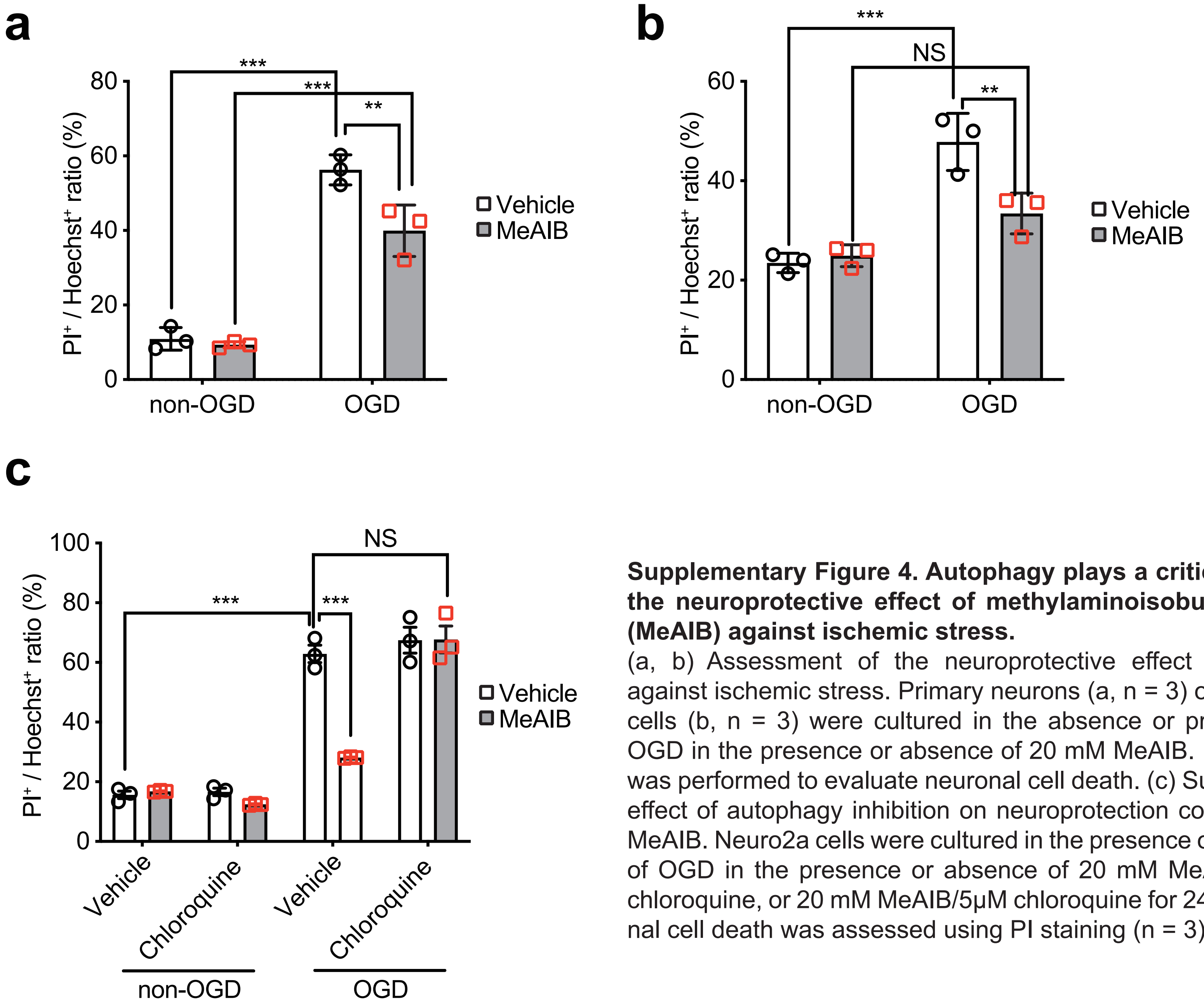

Supplementary Figure 4. Autophagy plays a critical role in the neuroprotective effect of methylaminoisobutyric acid (MeAIB) against ischemic stress.
(a, b) Assessment of the neuroprotective effect of MeAIB against ischemic stress. Primary neurons (a, n = 3) or Neuro2a cells (b, n = 3) were cultured in the absence or presence of OGD in the presence or absence of 20 mM MeAIB. PI staining was performed to evaluate neuronal cell death. (c) Suppressive effect of autophagy inhibition on neuroprotection conferred by MeAIB. Neuro2a cells were cultured in the presence or absence of OGD in the presence or absence of 20 mM MeAIB, 5 μM chloroquine, or 20 mM MeAIB/5μM chloroquine for 24 h. Neuronal cell death was assessed using PI staining (n = 3).

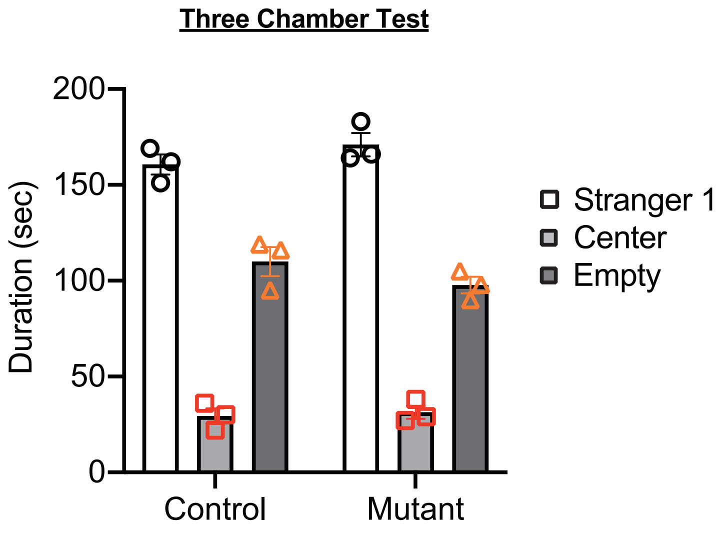

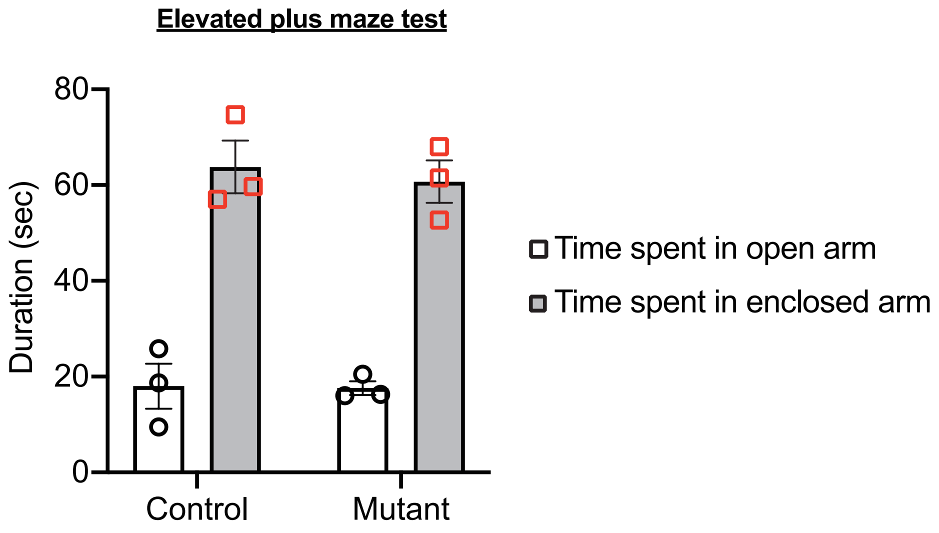

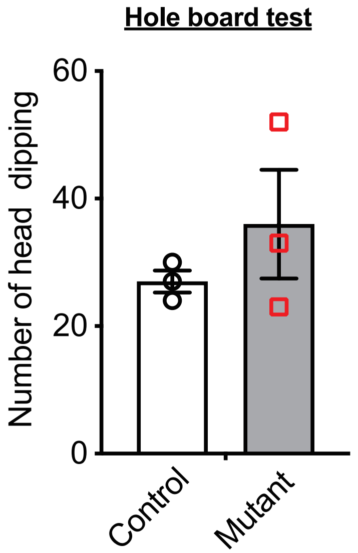

Supplementary Figure 5. Behavioral tests of mutant mice
Three chamber test, elevated plus maze test and hole board test were performed to investigate the effect of Slc38a1 deficiency on behaviors. 6 week-old male mice were used but there were no differences (n = 3 in each test)

a

Neurological symptom		Score
Ⅰ.Posture test	ⅰ) Normal	0
	ⅱ) Flexion of the right forelimb when raised by the tail (<90°)	1
	ⅲ) Flexion of the right forelimb when raised by the tail (>90°)	2
	ⅳ) Abnormal body posture (Bending and twisting posture) when raised by the tail	3
Ⅱ.Rotation test	ⅰ) No rotation	0
	ⅱ) Clockwise rotation when raised by the tail while the forelimb attaching to the floor	1
	ⅲ) Clockwise rotation when freely moving	2

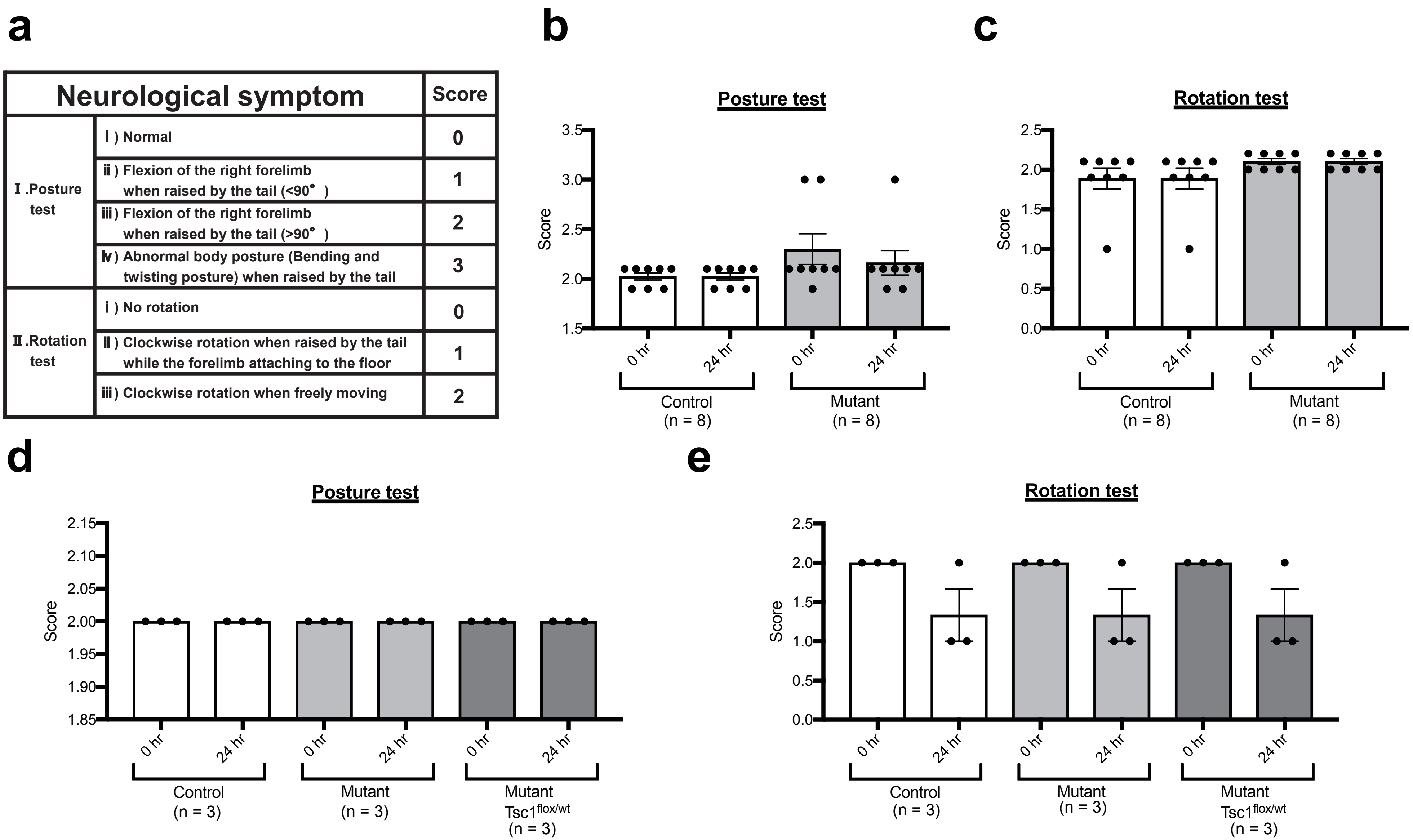

Supplementary Figure 6. Neurobehavioral assessments related to Fig.3 and Fig.4

(a) Neurological symptom score used in this study. (b, c) Exclusion criteria in Fig.3. Neurological symptoms were assessed 24 hours after surgery by posture or rotation test. Mice that had more than score 1 in either test were used for further experiments. (d, e) Exclusion criteria in Fig.4. Neurological symptoms were assessed by posture or rotation test 24 hours after surgery. Mice that had more than score 1 in either test were used for further experiments.

Fig. 2b

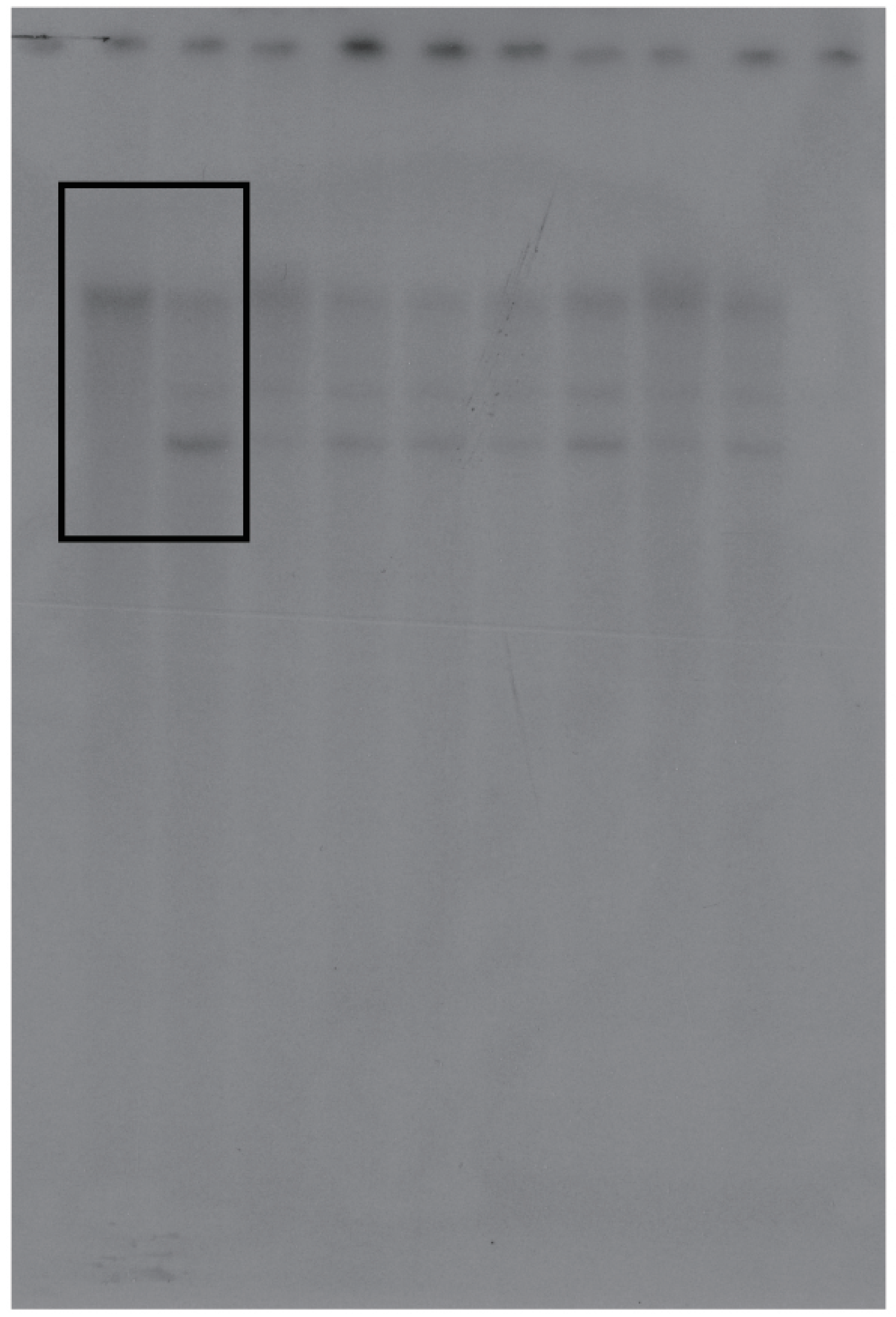

Fig. 2e (SNAT1)

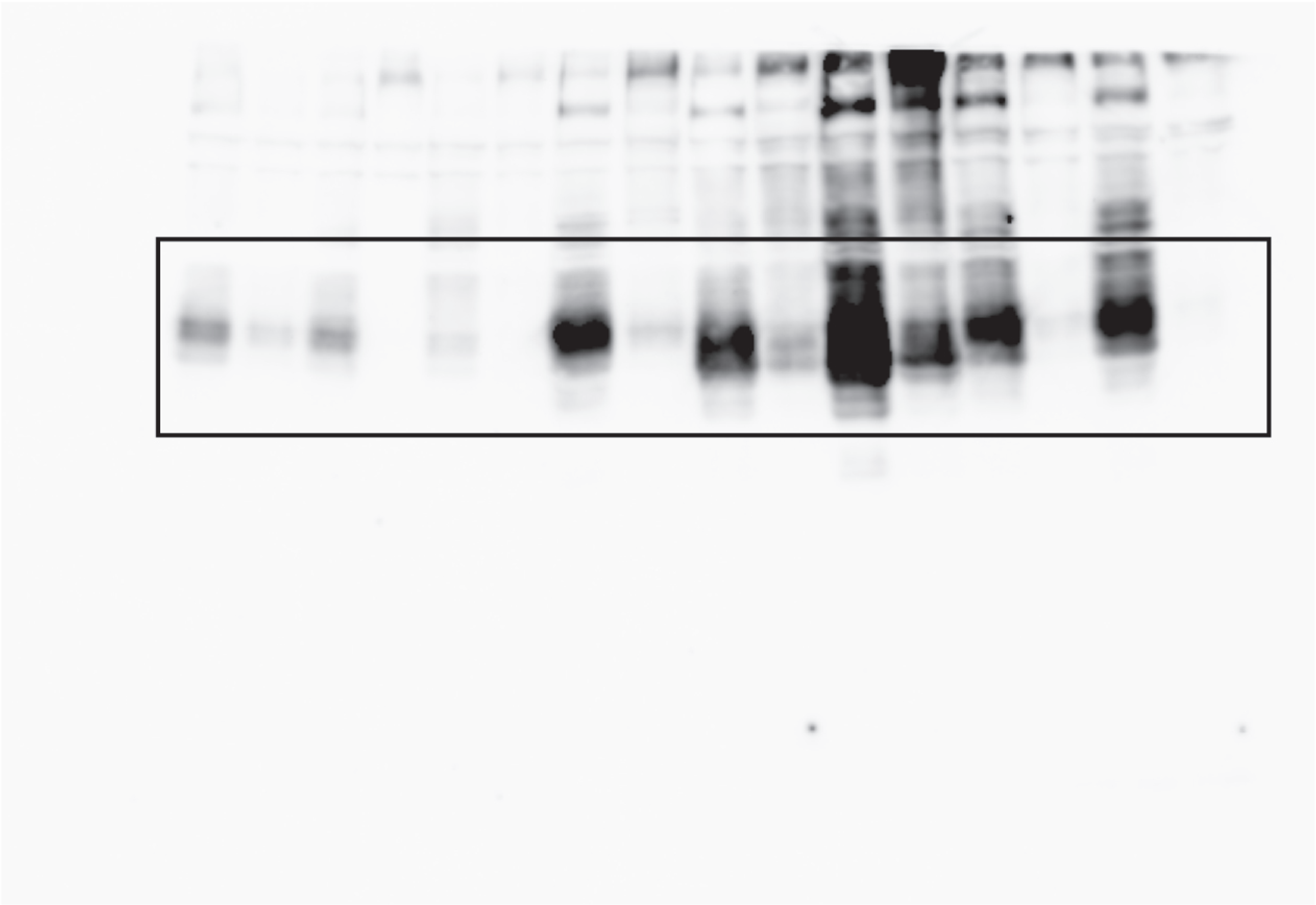

Fig. 2e (CBB)

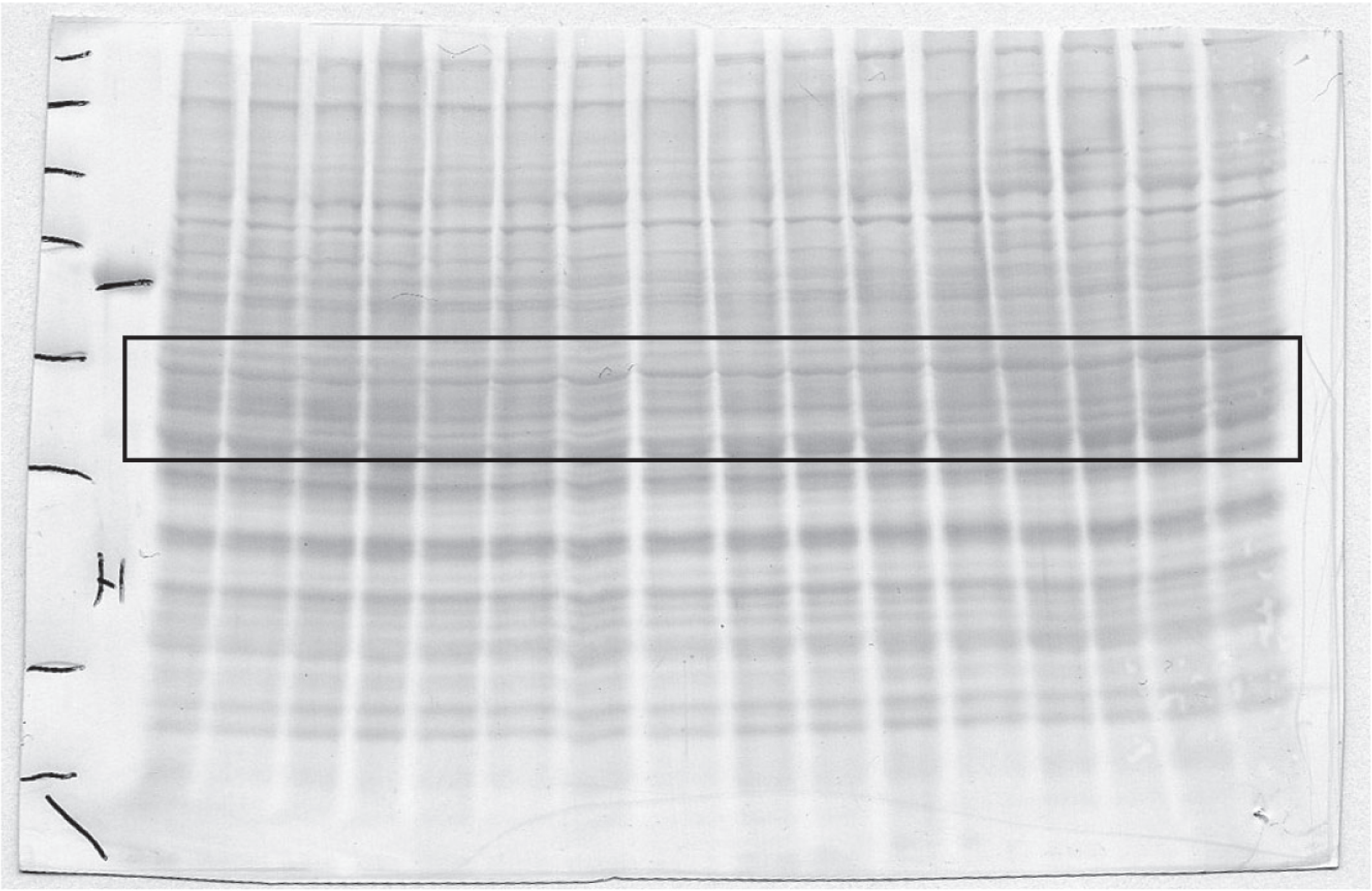

Fig. 5c (SNAT1)

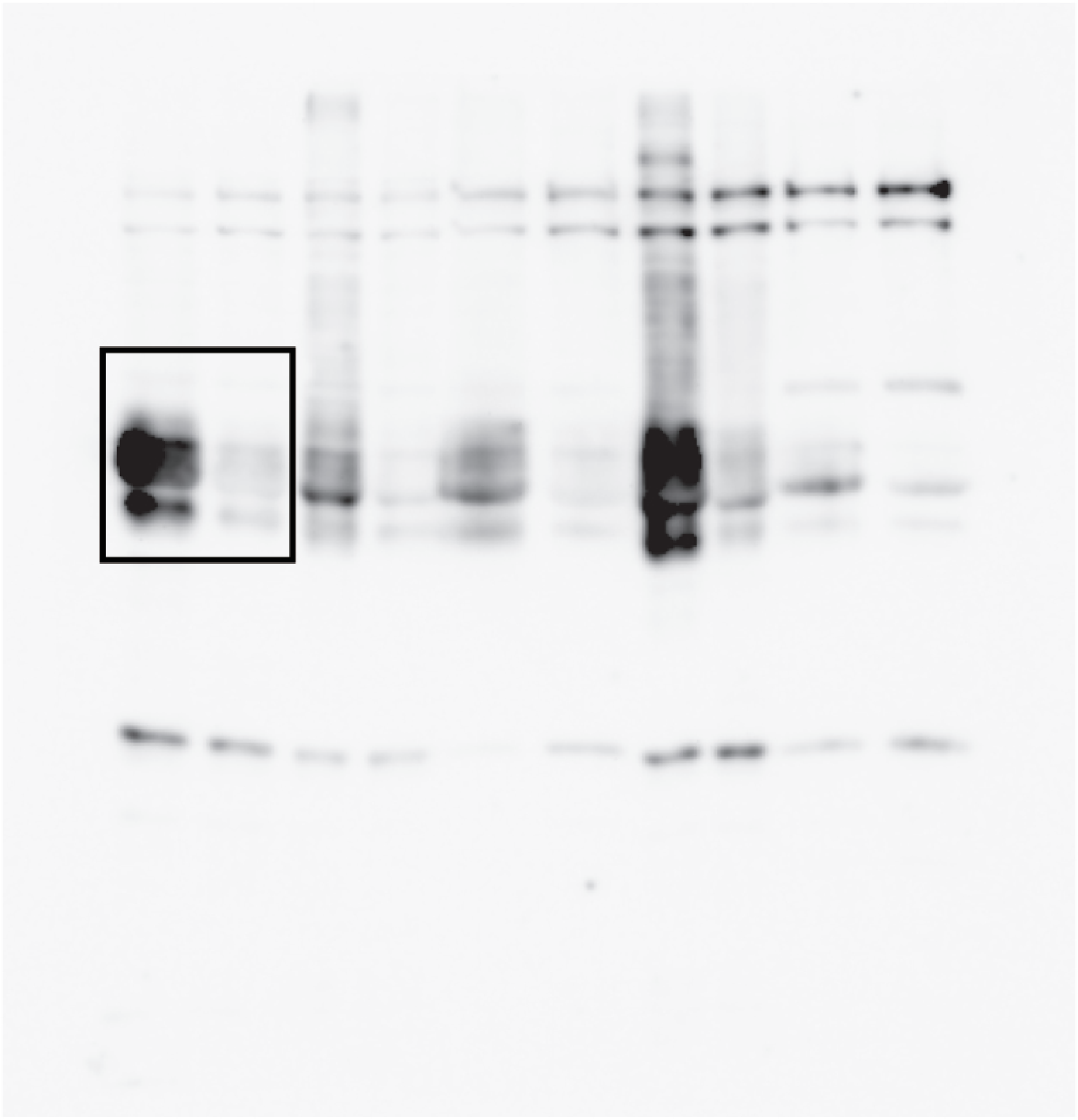

Fig. 5c (CBB)

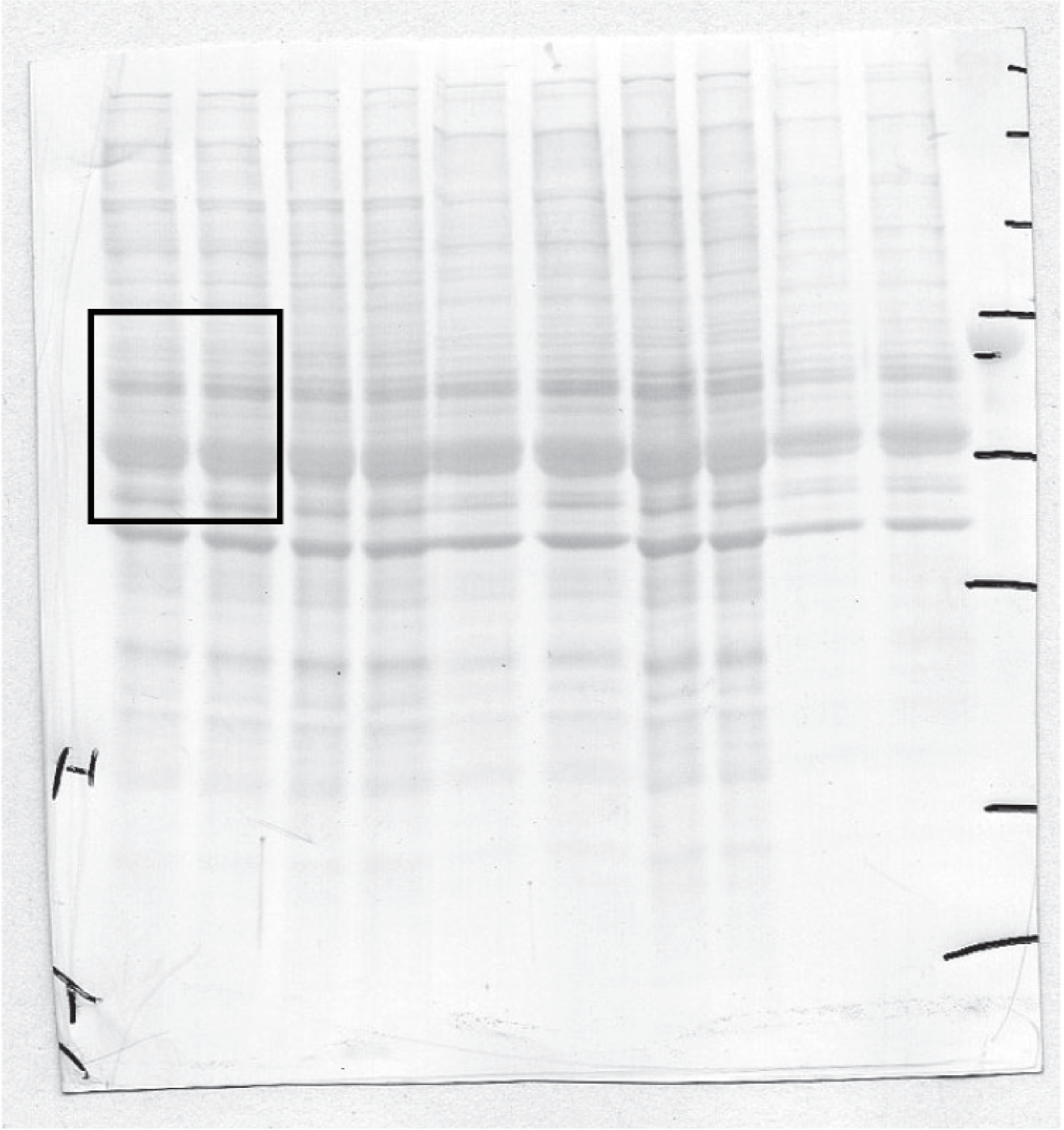

Supplementary Fig. 2c (SNAT1)

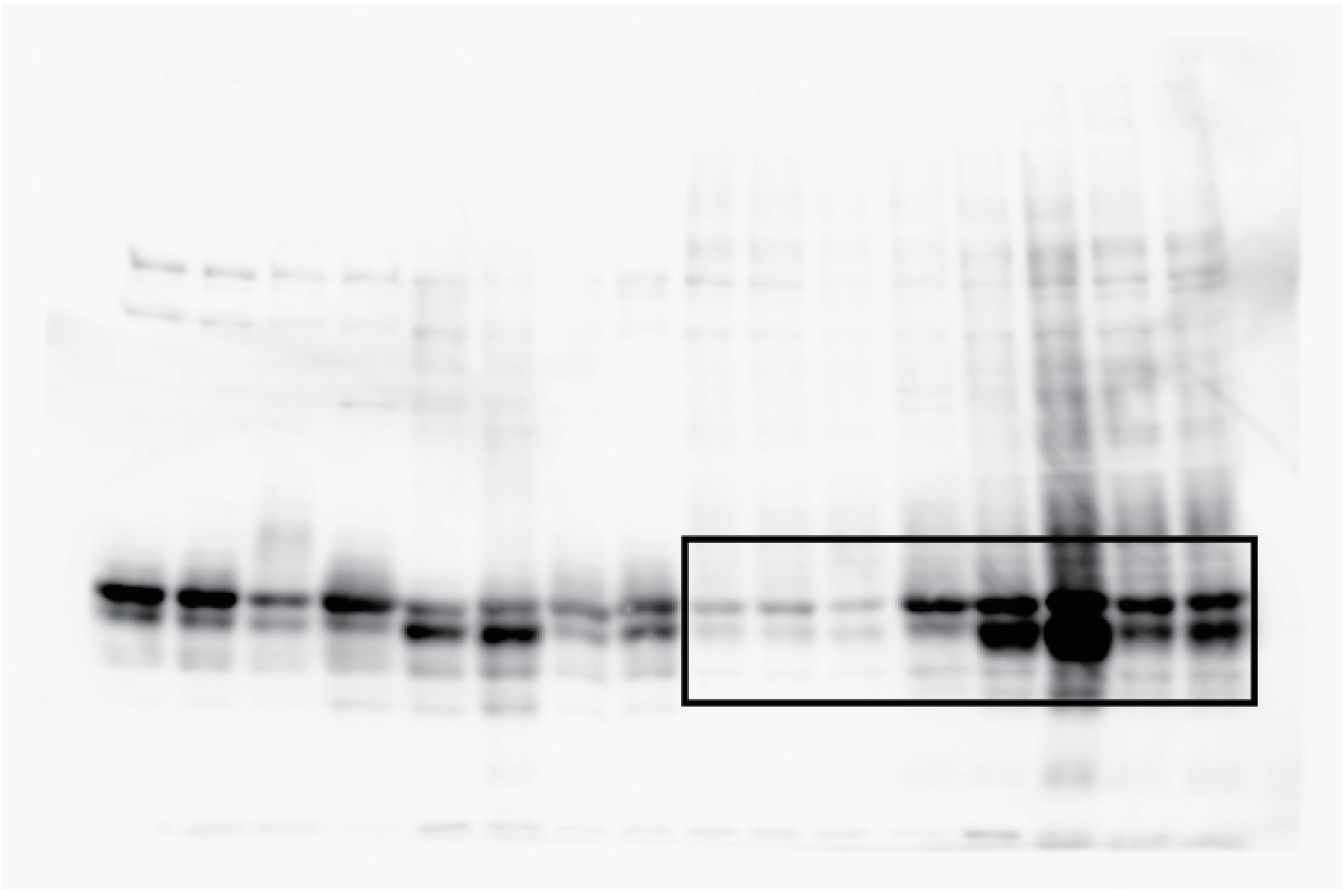

Supplementary Fig. 2c (CBB)

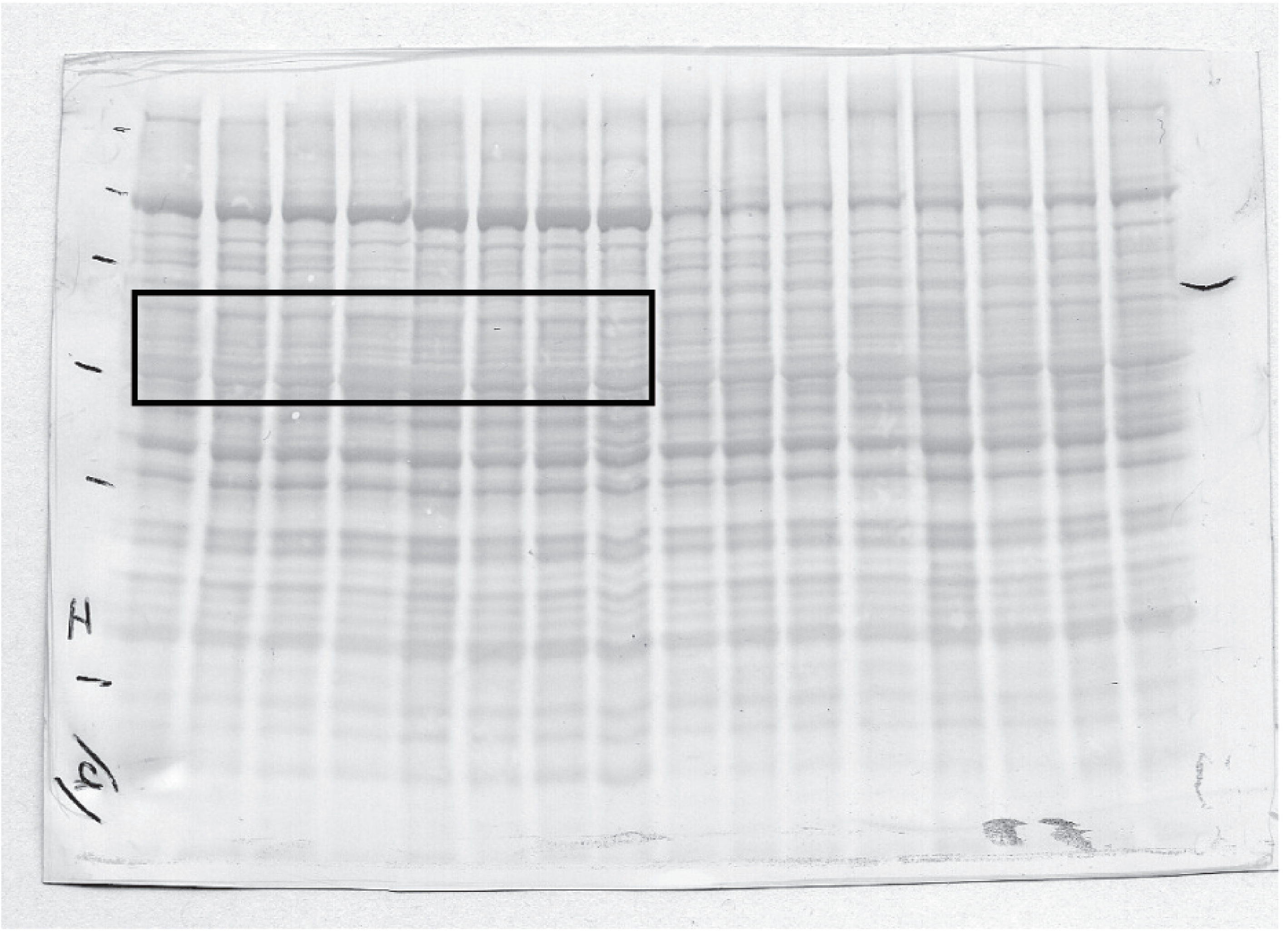

Supplementary Figure 7. Full blots shown in Fig. 2b, Fig. 2e, Fig. 5c and Supplementary Fig. 2c.

Supplementary Table 1. List of quantitative PCR primers

Gene	Upstream (5′-3′)	Downstream (5′-3′)	Product size (bp)	Annealing temperature (°C)
Gapdh	AGGTCGGTGTGAACGGATTTG	TGTAGACCATGTAGTTGAGGTCA	123	62
Actb	TGTGATGGTGGGAATGGGTCAGAA	TGTGGTGCCAGATCTTCTCCATGT	140	62
Slc38a1	GGCATCTGTATTTGCTGCTG	CGTTGCTGACGTTGTCATCT	163	62
Slc38a2	TAATCTGAGCAATGCGATTGTGG	AGATGGACGGAGTATAGCGAAAA	129	62
Slc38a3	GGAGGGGCTTCTACCAGTG	GGAAAAGGATGATGCCCGTATTG	239	62
Slc38a4	GCGGGGACAGTATTCAGGAC	GGAACTTCTGACTTTCGGCAT	102	62
Slc38a5	CAACCTCAGCAACGCTATCAT	AGACAGGAGAGCAATGCACAG	112	62
Slc38a8	TCCTCAGAGTGATCGGGGAC	GACAGGGGAAAGATGACCAGC	130	62
Slc7a5	CTGGATCGAGCTGCTCATC	GTTCACAGCTGTGAGGAGC	160	62
Slc7a8	TCAGCGCCTGTGGTATCATTG	TGATGCCTGTCACGATCCAGA	126	62
Slc1a5	TTCGCTATCGTCTTTGGTGTG	ATGGTGGCATCATTGAAGGAG	92	62
Map1lc3	TTATAGAGCGATACAAGGGGGAG	CGCCGTCTGATTATCTTGATGAG	109	62
Lamp1	CAGCACTCTTTGAGGTGAAAAAC	ACGATCTGAGAACCATTCGCA	103	62
Sqstm1	AGGATGGGGACTTGGTTGC	TCACAGATCACATTGGGGTGC	178	62
Ctsb	TCCTTGATCCTTCTTTCTTGCC	ACAGTGCCACACAGCTTCTTC	176	62
Ctsd	GCTTCCGGTCTTTGACAACCT	CACCAAGCATTAGTTCTCCTCC	113	62

Supplementary Table 2. Genotyping primers

Mouse	Upstream (5′-3′)	Downstream (5′-3′)
Slc38a1$^{flox/flox}$ mouse (floxed allele)	AGATGACAACGTCAGCAACG	AAGGCTTTGAGACGTGTTGG
Slc38a1$^{flox/flox}$ mouse (deleted allele)	TGCTGCTAACTTGGGGTTTC	TTAAAATGTGGGCAATGGT
SynI-Cre mouse (cre recombinase)	GCATTACCGGTCGATGCAAC	TGAGTGAACGAACCTGGTCG
Tsc1$^{flox/flox}$ mouse (floxed allele)	AGGAGGCCTCTTCTGCTACCACTTTTGATG	GAAGGCAGCTCCGACCATGAAGTGCTGTGT
