# Supplementary material for: Inhibition of the glutamine transporter SNAT1 confers neuroprotection in mice by modulating the mTOR-autophagy system
Source: Commun Biol. 2019 Sep 18;2:346. doi: 10.1038/s42003-019-0582-4 (PMC6751179; doi:10.1038/s42003-019-0582-4)
Supplement: Supplementary file 2 — Description of Additional Supplementary Files [file 42003_2019_582_MOESM2_ESM.docx]

**Description of Additional Supplementary Files**

**File Name**: Supplementary Data

**Description**: Source data
