# Supplementary material for: Inhibition of the glutamine transporter SNAT1 confers neuroprotection in mice by modulating the mTOR-autophagy system
Source: Commun Biol. 2019 Sep 18;2:346. doi: 10.1038/s42003-019-0582-4 (PMC6751179; doi:10.1038/s42003-019-0582-4)
Supplement: Supplementary file 4 — Reporting Summary [file 42003_2019_582_MOESM4_ESM.pdf]

nature research

Corresponding author(s): Takeshi Takarada

Last updated by author(s): Jul 28, 2019

# Reporting Summary

Nature Research wishes to improve the reproducibility of the work that we publish. This form provides structure for consistency and transparency in reporting. For further information on Nature Research policies, see Authors & Referees and the Editorial Policy Checklist .

## Statistics

For all statistical analyses, confirm that the following items are present in the figure legend, table legend, main text, or Methods section.

| n/a | Confirmed | |
|---|---|---|
| ☐ | ☒ | The exact sample size ($n$) for each experimental group/condition, given as a discrete number and unit of measurement |
| ☐ | ☒ | A statement on whether measurements were taken from distinct samples or whether the same sample was measured repeatedly |
| ☐ | ☒ | The statistical test(s) used AND whether they are one- or two-sided<br>*Only common tests should be described solely by name; describe more complex techniques in the Methods section.* |
| ☒ | ☐ | A description of all covariates tested |
| ☐ | ☒ | A description of any assumptions or corrections, such as tests of normality and adjustment for multiple comparisons |
| ☐ | ☒ | A full description of the statistical parameters including central tendency (e.g. means) or other basic estimates (e.g. regression coefficient) AND variation (e.g. standard deviation) or associated estimates of uncertainty (e.g. confidence intervals) |
| ☐ | ☒ | For null hypothesis testing, the test statistic (e.g. $F$, $t$, $r$) with confidence intervals, effect sizes, degrees of freedom and $P$ value noted<br>*Give P values as exact values whenever suitable.* |
| ☒ | ☐ | For Bayesian analysis, information on the choice of priors and Markov chain Monte Carlo settings |
| ☒ | ☐ | For hierarchical and complex designs, identification of the appropriate level for tests and full reporting of outcomes |
| ☒ | ☐ | Estimates of effect sizes (e.g. Cohen's $d$, Pearson's $r$), indicating how they were calculated |

*Our web collection on statistics for biologists contains articles on many of the points above.*

## Software and code

Policy information about availability of computer code

| | |
|---|---|
| Data collection | MiniOpticon real-time PCR system (BioRad) and CFX Manager Software v3.1 (BioRad) was used for data acquisition of quantitative PCR. Confocal images were acquired with LSM710 microscope (Zeiss) and ZEN2009 software (Zeiss). LAS-3000 (GE Healthcare) was used for data acquisition of western blot analyses. BZ-X700 (keyence) was used to acquire microscopic images for TTC or DAB stain. |
| Data analysis | ImageJ 1.49v (National Institute of Health) was used for data analyses of western blot images. In Cell Analyzer 2000 (GE Healthcare) was used for image analyses in confocal laser scanning microscopy. CFX Manager Software v3.1 (BioRad) was used for data analyses of quantitative PCR. GraphPad Prism 7 (GraphPad Software) was used for other data calculations and statistical analyses. |

For manuscripts utilizing custom algorithms or software that are central to the research but not yet described in published literature, software must be made available to editors/reviewers. We strongly encourage code deposition in a community repository (e.g. GitHub). See the Nature Research guidelines for submitting code & software for further information.

## Data

Policy information about availability of data

All manuscripts must include a data availability statement. This statement should provide the following information, where applicable:

- Accession codes, unique identifiers, or web links for publicly available datasets
- A list of figures that have associated raw data
- A description of any restrictions on data availability

Source Data for the figures and supplementary figures including their descriptions are provided in the online version of this paper.

# Field-specific reporting

Please select the one below that is the best fit for your research. If you are not sure, read the appropriate sections before making your selection.

☒ Life sciences ☐ Behavioural & social sciences ☐ Ecological, evolutionary & environmental sciences

For a reference copy of the document with all sections, see nature.com/documents/nr-reporting-summary-flat.pdf

# Life sciences study design

All studies must disclose on these points even when the disclosure is negative.

| | |
|---|---|
| Sample size | Although no statistical methods were used to predetermine sample size in vitro and in vivo analyses, we conducted preliminary experiments to estimate variances in each assay and determined sufficient sample size. |
| Data exclusions | In MCAO experiments, neurobehavioral assessments, including posture and rotation tests (blinded), were performed after the recovery time and animals with neurobehavioral defects were used for subsequent experiments. However, we used all mice that experienced MCAO surgery and found all of them had some defects in both tests. |
| Replication | We successfully reproduced all data shown in our manuscript. |
| Randomization | All animal experiments were performed using mice of the same age and similar weights. Thus, we did not perform randomization. |
| Blinding | To perform histomorphometric or neurobehavioal analyses, the data analyzer was blinded to group allocation. |

# Reporting for specific materials, systems and methods

We require information from authors about some types of materials, experimental systems and methods used in many studies. Here, indicate whether each material, system or method listed is relevant to your study. If you are not sure if a list item applies to your research, read the appropriate section before selecting a response.

## Materials & experimental systems

| n/a | Involved in the study | |
|---|---|---|
| ☐ | ☒ | Antibodies |
| ☐ | ☒ | Eukaryotic cell lines |
| ☒ | ☐ | Palaeontology |
| ☐ | ☒ | Animals and other organisms |
| ☒ | ☐ | Human research participants |
| ☒ | ☐ | Clinical data |

## Methods

| n/a | Involved in the study | |
|---|---|---|
| ☒ | ☐ | ChIP-seq |
| ☒ | ☐ | Flow cytometry |
| ☒ | ☐ | MRI-based neuroimaging |

## Antibodies

| | |
|---|---|
| Antibodies used | SNAT1 (kindly provided by Dr. Jeffrey D. Erickson, 1:200 for IHC, 1:2,000 for WB), NeuN (MAB377 Chemicon, 1:400 for IHC), S100β (S2532 SIGMA, 1:400 for IHC), CD11b (MCA711G Serotec, 1:200 for IHC), MAP2 (M4403, SIGMA, 1:200 for IHC or ICC), phospho-p70 S6 kinase (Thr389) (#9234 Cell Signaling Technology, 1:200 for IHC, 1:2,000 for WB), p70 S6 kinase (#2708 Cell Signaling Technology, 1:2,000 for WB), phospho-mTOR (Ser2448) (#2971 Cell Signaling Technology, 1:2,000 for WB), phospho-S6 (Ser235/236) (#4858 Cell Signaling Technology, 1:2,000 for WB), phospho-Akt (Ser473) (#4060 Cell Signaling Technology, 1:2,000 for WB), GAPDH (sc-25778 Santa Cruz, 1:2,000 for WB), β-tubulin (T4026 Sigma, 1:2,000 for WB) and β-actin (sc-4778 Santa Cruz, 1:2,000 for WB), anti-rabbit IgG antibody conjugated with Alexa 594 (A-11012 Invitrogen, 1:400 for IHC), anti-mouse IgG antibody conjugated with Alexa 488 (A-11011 Invitrogen, 1:400 for IHC or ICC), anti-rat IgG antibody conjugated with Alexa 488 (A-11006 Invitrogen, 1:400 for IHC), anti-mouse IgG antibody conjugated with HRP (#7074 Cell Signaling Technology, 1:5,000 for WB), anti-rabbit IgG antibody conjugated with HRP (#7076 Cell Signaling Technology, 1:5,000 for WB) |
| Validation | SNAT1 (kindly provided by Dr. Jeffrey D. Erickson), NeuN (MAB377 Chemicon), S100β (S2532 SIGMA), CD11b (MCA711G Serotec), MAP2 (M4403, SIGMA), phospho-p70 S6 kinase (Thr389) (#9234 Cell Signaling Technology), p70 S6 kinase (#2708 Cell Signaling Technology), phospho-mTOR (Ser2448) (#2971 Cell Signaling Technology), phospho-S6 (Ser235/236) (#4858 Cell Signaling Technology), phospho-Akt (Ser473) (#4060 Cell Signaling Technology), GAPDH (sc-25778 Santa Cruz), β-tubulin (T4026 Sigma) and β-actin (sc-4778 Santa Cruz), anti-rabbit IgG antibody conjugated with Alexa 594 (A-11012 Invitrogen), anti-mouse IgG antibody conjugated with Alexa 488 (A-11011 Invitrogen), anti-rat IgG antibody conjugated with Alexa 488 (A-11006 Invitrogen), anti-mouse IgG antibody conjugated with HRP (#7074 Cell Signaling Technology), anti-rabbit IgG antibody conjugated with HRP (#7076 Cell Signaling Technology) |

## Eukaryotic cell lines

Policy information about cell lines

| | |
|---|---|
| Cell line source(s) | HEK293T - RIKEN Cell Bank, Neuro2a - ATCC |
| Authentication | All cell lines used in this study were authenticated by the distributors. |
| Mycoplasma contamination | We confirmed that all cell lines were negative for mycoplasma contamination. |
| Commonly misidentified lines (See ICLAC register) | Any cells used in this study are not listed in ICLAC database. |

## Animals and other organisms

Policy information about studies involving animals; ARRIVE guidelines recommended for reporting animal research

| | |
|---|---|
| Laboratory animals | To prepare RNA samples, 6-week-old male C57BL/6 mice (Fig.1a-b, Supplementary Fig.1a) or 6-week-old male mutant littermates (Fig.2d) were used. For immunohistochemical analysis, 6-week-old C57BL/6 mice (Fig.1c) or 6-week-old male mutant littermates (Fig.2f, Fig.3, Fig.4) were used. For in situ hybridization assay (SupplementaryFig.1b), 6-week-old male C57BL/6 mice were used. To prepare western blot samples, 6-week-old male mutant littermates (Fig.2e, Fig.4d) or 6-week-old C57BL/6 mice (Supplementary Fig.1c) were used. To isolate and culture primary neurons, E15 ddY mice (Supplementary Fig.3b-e, Supplementary Fig.4a-c) and E15 mutant mice (Fig.5, Fig.6, Supplementary Fig.3a) were used. |
| Wild animals | Any wild animal were not used in this study. |
| Field-collected samples | Samples collected from the field were not used in this study. |
| Ethics oversight | All animal experiments were approved by the Committee on Animal Experimentation of Kanazawa University and Okayama University and performed in compliance with the University's Guidelines for the Care and Use of Laboratory Animals. |

Note that full information on the approval of the study protocol must also be provided in the manuscript.
